# Phenotypic and Transcriptional Fidelity of Patient-Derived Colon Cancer Xenografts in Immune-Deficient Mice

**DOI:** 10.1371/journal.pone.0079874

**Published:** 2013-11-20

**Authors:** Jeffrey Chou, Matthew P. Fitzgibbon, Christie-Lynn L. Mortales, Andrea M. H. Towlerton, Melissa P. Upton, Raymond S. Yeung, Martin W. McIntosh, Edus H. Warren

**Affiliations:** 1 Program in Immunology, Clinical Research Division, Fred Hutchinson Cancer Research Center, Seattle, Washington, United States of America; 2 Division of Medical Oncology, Department of Medicine, University of Washington Medical Center, Seattle, Washington, United States of America; 3 Computational Biology Program, Public Health Sciences Division, Fred Hutchinson Cancer Research Center, Seattle, Washington, United States of America; 4 Gastrointestinal and Liver Pathology Service, Department of Pathology, University of Washington, Seattle, Washington, United States of America; 5 Department of Surgery, University of Washington, Seattle, Washington, United States of America; University of Hong Kong, Hong Kong

## Abstract

Xenografts of human colorectal cancer (CRC) in immune-deficient mice have great potential for accelerating the study of tumor biology and therapy. We evaluated xenografts established in NOD/scid/IL2Rγ-null mice from the primary or metastatic tumors of 27 patients with CRC to estimate their capacity for expanding tumor cells for *in vitro* studies and to assess how faithfully they recapitulated the transcriptional profile of their parental tumors. RNA-seq analysis of parental human CRC tumors and their derivative xenografts demonstrated that reproducible transcriptional changes characterize the human tumor to murine xenograft transition. In most but not all cases, the human stroma, vasculature, and hematopoietic elements were systematically replaced by murine analogues while the carcinoma component persisted. Once established as xenografts, human CRC cells that could be propagated by serial transplantation remained transcriptionally stable. Three histologically atypical xenografts, established from patients with peritoneal metastases, contained abundant human stromal elements and blood vessels in addition to human tumor cells. The transcriptomes of these mixed tumor/stromal xenografts did not closely resemble those of their parental tumors, and attempts to propagate such xenografts by serial transplantation were unsuccessful. Stable expression of numerous genes previously identified as high priority targets for immunotherapy was observed in most xenograft lineages. Aberrant expression in CRC cells of human genes that are normally only expressed in hematopoietic cells was also observed. Our results suggest that human CRC cells expanded in murine xenografts have great utility for studies of tumor immunobiology and targeted therapies such as immunotherapy but also identify potential limitations.

## Introduction

Freshly resected primary and metastatic colorectal cancers (CRC) [1], [2], like many other types of human hematologic and solid tumors, can be established as xenografts in immune-deficient mice such as the NOD/*scid*/IL2Rγ−/− (NSG) strain and propagated long-term via serial transplantation. CRC xenografts provide an attractive model system in which to study tumor biology and therapy. In contrast to CRC cell lines established from fresh surgical tissues often lose important characteristics of their parental human tumors once they have adapted to, and been propagated in, *in vitro* culture, patient-derived CRC xenografts reproduce many of the phenotypic and genotypic features of the parental tumors from which they are derived [1]–[17]. Analysis of patient-derived CRC xenografts has profoundly advanced our understanding of tumor architecture, heterogeneity, and other critical aspects of tumor biology. CRC xenografts also have excellent potential for use as model systems in which to explore therapy with chemotherapeutic agents [9]–[13], [17] as well as novel forms of targeted CRC treatment such as immunotherapy [11], [17], [18]. In addition, the ability to propagate human CRC in immune-deficient mice can potentially provide a renewable supply of CRC cells for use in *in vitro* studies.

Differences between human CRC tumors and their derivative xenografts, however, do exist. Most significant is the consistent observation that CRC xenografts are supported by murine, rather than human, stroma [7], [9], [15], [16]. Consequently, CRC xenografts offer an opportunity to evaluate the transcriptome of human carcinoma cells separately from that of their supporting human stroma. Deep transcriptional analysis of CRC xenografts can therefore aid with the interpretation of published transcriptional analyses of freshly resected human CRC tumors, which represent a mixture of both human stroma and carcinoma [19], [20]. The extent to which interaction with murine stroma and vasculature influences the transcriptional profile of the human tumor cells in the xenograft has not been examined to date with methods that permit unambiguous discrimination between human transcripts derived from the tumor cells and murine transcripts derived from the stroma and vasculature. We therefore undertook a comprehensive study of the morphology and transcriptional profiles of a panel of xenografts established in NSG mice from freshly resected primary and metastatic human colon cancers, to evaluate the fidelity with which the xenografts recapitulated the transcriptional profiles and unique molecular features of the parental human tumors from which they were derived. The transcriptional analysis was performed with RNA-seq, which permits determination of the human or murine origin of transcripts with a high degree of confidence. To assess the stability of these features through serial transplantation, we also performed detailed transcriptional analysis of a xenograft lineage that was established from a single primary human colon tumor and has subsequently been propagated through ten generations of NSG recipients. We determined that xenotransplantation of human colon tumors is characterized by reproducible biologic and transcriptional changes that characterize the human to murine transition, which in most cases select for a transcriptionally stable tumor population supported by murine stroma and vasculature. In addition, we identified gene sets selectively expressed in the epithelial (carcinoma) or stromal components of CRC xenografts.

## Materials and Methods

### Ethics Statement

Surgical samples of primary or metastatic colorectal cancer were obtained from de-identified donors through the Cooperative Human Tissue Network (CHTN, Vanderbilt University, Nashville, TN), or from subjects being treated at the University of Washington (UW) Medical Center (Seattle, WA) who gave written informed consent according to a protocol approved by the Institutional Review Board of the UW/Fred Hutchinson Cancer Research Center (FHCRC) Cancer Consortium. Investigation of human samples was conducted according to the principles expressed in the Declaration of Helsinki. This study was carried out in strict accordance with the recommendations found in the Guide for the Care and Use of Laboratory Animals, 8^th^ Edition (National Research Council, National Academies Press). The protocol was approved by the Institutional Animal Care and Use Committee of the FHCRC. All surgery was performed under tribromoethanol anesthesia, and all efforts were made to minimize suffering.

### Transport and Processing of Tissue Samples

Freshly resected metastatic tumor and/or primary tumor colorectal cancer specimens obtained from the CHTN (23 unique patients, 24 specimens) or locally (27 unique patients, 33 specimens) were placed in transport medium consisting of DMEM/F12 medium (Hyclone) supplemented with 10 µg/mL ciprofloxacin (Bayer), 1% penicillin/streptomycin (10,000 U/mL penicillin, 10 mg/mL streptomycin) (Invitrogen), and 0.5 µg/mL amphotericin B (Invitrogen). They were shipped overnight on ice for processing the following day (CHTN tumors) or transported within one hour for processing on the same day (UW tumors). Small portions of each tumor were flash frozen or placed in RNAlater (Life Technologies) and also placed in formalin. The balance of each tumor was rinsed with phosphate buffered saline (Invitrogen), then reduced to a single cell suspension via mechanical disruption, and enzymatic digestion over 1–2 hours in the DMEM/F12-based transport medium supplemented with 4.66 µg/mL heparin sodium (Sigma), 2% B27 supplement (Invitrogen), and 5% KnockOut Serum Replacement (Invitrogen), 1 mg/mL Collagenase I (Sigma), 0.1 mg/mL Hyaluronidase, and 0.1 mg/mL DNase I (Worthington Biochemical). Digested cells were washed with enzyme-free medium and resuspended in 30–50 µL Matrigel (BD Biosciences) prior to injection.

Primary xenografts were established by injecting single cell suspensions (1×10^4^–2×10^6^ cells) prepared from colorectal cancer specimens either under the kidney capsule [21] (from n = 14 unique patients) and/or subcutaneously in the flanks (n = 46 patients) of 6–12 week old male or female sublethally γ-irradiated (250 cGy at 20 cGy/min) NSG mice raised at the Fred Hutchinson Cancer Research Center. The interval between surgical resection and mouse injection was 24–30 hours for colon tumors obtained from the CHTN and 3–6 hours for tumors obtained locally. Five digested tumor samples were enriched for CD133^+^ cells prior to injection, using magnetic beads conjugated to anti-CD133 antibody (Miltenyi Biotec) as per the manufacturer’s directions. Mice bearing progressively enlarging tumors were allowed to survive until the tumors reached a diameter of 2 cm, they manifested any signs of suffering, or they sustained 20% weight loss, at which point they were euthanized for tumor harvest. Pieces of harvested xenograft were immediately flash frozen or placed in RNAlater, placed in formalin for subsequent histology, and placed in transport medium and processed in an identical fashion to the original human tumor for serial transplantation into another NSG host.

### Immunohistochemistry and Microscopy

Formalin-fixed, paraffin-embedded tissue sections from resected colon cancer samples or xenografts were prepared for microscopy by staining with hematoxylin and eosin, or with antibodies against human leukocyte antigen (HLA) class I (MBL Corporation), carcinoembryonic antigen (CEA) (Novus), cytokeratin 20 (CK20) (Dako), Ki-67 (Dako), CD31 (Dako), epithelial cell adhesion molecule (EPCAM) (Novus), E-cadherin (Cell Marque), Programmed Death-1 (PD1) (Cell Marque), vimentin (Dako), fibronectin (Dako), CD4 (Novacastra), CD8 (Novacastra), and CD3 (Novacastra) as per the manufacturers’ instructions. Positive and negative controls were performed for each antibody. Slides were viewed on a Nikon E800 Eclipse microscope, and images were acquired with an Olympus Magnafire SP camera and Magnafire software (Optronics).

### Flow Cytometry

A single cell suspension prepared from a xenograft derived from D61540.T2 was stained with Qdot 525 nanocrystal (Invitrogen) for live and dead cell differentiation, fluorescein isothiocyanate (FITC)-conjugated anti-PD-1 antibody (BD Biosciences), and phycoerythrin (PE)-conjugated anti-CTLA-4 antibody (BD Biosciences). Data were acquired on a FACSCanto II flow cytometer (BD Biosciences) and analyzed with Kaluza software (Beckman Coulter).

### RNA-seq Sample Preparation and Sequencing

RNA was extracted from samples using RNeasy Plus Mini or AllPrep RNA/DNA kits (QIAGEN). The quality of RNA was assessed on an Agilent 2100 Bioanalyzer. Samples with RNA integrity number (RIN) greater than 7 were diluted to 50 ng/µL for sequencing library preparation with the TruSeq Sample Preparation Kit (Illumina). Starting with approximately 1 µg of total RNA, mRNA was isolated with oligo-dT capture beads, then fragmented and converted to cDNA with random hexamer-primed reverse transcription and second-strand synthesis. Resulting cDNAs were fragmented by sonication and size-selected for molecules of ∼300 bp. Ligation of barcoded sequencing adapters was then performed according to manufacturer’s recommendations. The cDNA samples underwent multiplex sequencing, with one to four samples per lane, on the Illumina HiSeq 2000 to yield 50 bp paired-end sequences. This process yielded between 54.6 M and 367.0 M sequences passing the default Illumina quality control filters.

### Quantitative PCR

Cryopreserved cells from P2726.Ov were processed with a Dead Cell Removal Kit (Miltenyi Biotec) and then sorted into EPCAM^−^ and EPCAM^+^ fractions with magnetic beads conjugated to human EPCAM-specific antibody (Miltenyi Biotec). RNA was extracted from each fraction, and cDNA was prepared using oligo-dT and random hexamer primers from the First Strand cDNA Synthesis kit (Roche). The 5′ and 3′ primers for quantitative PCR (qPCR) were designed using either qPrimerDepot [22] or Primer-BLAST [23] (Table S1 in File S4). Standard SYBR Green (Roche) qPCR performed on an ABI Prism 7900HT was used to detect gene transcripts. The fluorescence data were analyzed in LinRegPCR [24], and the estimated starting concentration (N_0_) of each gene was normalized against that of housekeeping gene *GAPDH*. The human origin of PCR amplicons from xenografts was verified by dissociation curve analysis.

### RNA-seq Data Analysis

For each sample, all paired reads passing the quality filters were processed with the TopHat splice-aware short-read aligner [25]. For the xenograft samples, we employed a simple conservative filtering strategy to separate human reads, arising from the engrafted human tumor cells, from mouse reads expected to be sampled from supporting murine tissue. In this strategy, TopHat was used to align reads to both human (hg19) and mouse (mm9) reference genome assemblies. Reads that matched the human genome with higher fidelity (fewer mismatching bases) were retained as human reads. Those that matched the mouse genome with higher fidelity were retained as murine. Reads matching both genomes with equal fidelity were placed in a third “ambiguous” class and not considered further. To allow useful comparison between human tumor samples (free by construction from contaminating mouse sequences) and derived xenografts, we processed the human samples through the same filtering pipeline. Fewer than 0.2% of reads from these exclusively human samples were erroneously flagged as originating in mouse, suggesting that our filtering strategy has a low misclassification rate. Table 1 summarizes the sequencing yield, ranging from at least 5 GB to over 30 GB per sample, along with the percentage of reads in each sample classified as human, mouse, or ambiguous. Sequencing data are available in the NCBI Sequence Read Archive (accession #: SRP028952).

**Table 1:** RNA-seq sequencing yield and alignment to human (Hs19) or mouse (Mm9) genomes.

Sample	TotalSequences	HumanSequences	HumanReads (%)	MouseReads (%)	AmbiguousReads (%)	LanesSequenced
**D55949.X2**	187,239,646	178,775,171	95.48%	3.86%	0.66%	½
**D55949.X3F**	172,624,239	159,526,033	92.41%	6.92%	0.67%	½
**D55949.X3M**	147,142,165	137,838,437	93.68%	5.38%	0.95%	½
**D55949.X4**	85,188,100	75,005,625	88.05%	10.60%	1.35%	¼
**D55949.X7**	71,639,209	67,467,192	94.18%	3.14%	2.68%	¼
**P2750.Tu**	64,857,811	62,853,914	96.91%	0.15%	2.94%	¼
**P2750.Tu.X1**	90,871,024	52,366,808	57.63%	40.22%	2.15%	¼
**P2726.Om**	161,309,418	158,565,589	98.30%	0.13%	1.57%	½
**P2726.Ov**	151,183,879	149,007,052	98.56%	0.12%	1.32%	½
**P2726.Ov.X1**	83,880,021	79,448,364	94.72%	2.76%	2.52%	¼
**P2726.Ov.X2**	53,317,604	51,197,958	96.02%	1.84%	2.14%	¼
**P2726.As.X1**	86,050,324	66,038,390	76.74%	20.44%	2.81%	¼
**D61540.T1**	300,555,062	297,764,514	99.07%	0.10%	0.83%	1
**D61540.T2**	338,780,157	335,511,863	99.04%	0.09%	0.87%	1
**D61540.T2.X1**	184,556,372	174,663,794	94.64%	3.66%	1.70%	½
**D61540.T2.X2**	91,905,423	85,460,571	92.99%	4.14%	2.87%	¼

Sequence reads that matched the human genome with higher fidelity (fewer mismatched bases) were classified as human reads, and those that matched the mouse genome with higher fidelity were classified as murine. Reads matching the two genomes with equal fidelity were classified as “ambiguous” and not considered further.

Gene-level read counts were generated from human and mouse aligned reads using the HTSeq package (http://www-huber.embl.de/users/anders/HTSeq). Read counts were generated with the htseq-count script for each human and mouse gene locus in a union of the RefSeq and UCSC KnownGenes collections associated with the human hg19 and mm9 assemblies. Reads completely contained within any exon of a gene model were ascribed to that gene. The read counts for each gene were normalized by dividing them by the total number of reads, in millions, obtained for that sample.

### Statistical Analysis

Statistical analysis was performed in the R environment for statistical computing and Microsoft Excel. Unsupervised hierarchical cluster analysis of gene expression data was performed using the R environment for statistical computing. The package edgeR 3.0.2 [26] was used to identify genes that were differentially expressed in two or more samples. Tagwise dispersions were calculated for each gene using the generalized linear model method, and likelihood ratio tests were calculated between pairs of sample sets. Genes with false discovery rates of <0.05 after Benjamini-Hochberg adjustment [27] for multiple comparisons were defined as differentially expressed. Over- or under-represented gene sets amongst differentially expressed genes were identified using R package goseq 1.10.0 [28], with the canonical pathway and chemical and genetic perturbations gene sets from the Molecular Signatures Database (MSigDB) v3.0 [29]. The Wallenius non-central hypergeometric distribution method was used to approximate the distribution of gene set members amongst the differentially expressed genes.

Tumor growth curves were fitted to the equation 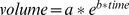 with doubling time defined as 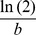 and goodness of fit measured by the coefficient of determination R^2^. The two-tailed Fisher’s exact test was used for comparisons of categorical data between two groups while two-tailed Student’s t-test was used for comparisons of quantitative data between two groups.

## Results

### Experience with Generating and Propagating CRC Xenografts

An advantage of xenotransplantation is that it potentially provides a method to propagate CRC cells indefinitely and faithfully replicates the original parental human tumor. We sought to develop a protocol that would most efficiently accomplish this goal. Sublethally irradiated NSG mice were injected with single cell suspensions prepared from tumor samples without prolonged intervening *in vitro* culture that could potentially select for adaptations not present in the original human tumor. A total of 33 of 57 surgical specimens obtained from 27 out of 50 individual patients with either locoregional and/or metastatic CRC (Table S2 in File S4) were successfully established as xenografts.

Initially, injection of CRC tumor cells enriched prior to implantation for cells expressing the putative CRC stem cell marker CD133^+^ was compared with injection of bulk, unsorted tumor cells for establishing xenografts because the former has been associated with a higher probability of engraftment [1], [2]. Whenever possible, the maximum number of CD133^+^-sorted cells or bulk, unsorted cells obtainable from a tumor was injected, at a dose of up to 2×10^6^ cells. Although injection of 1×10**^4^** CD133^+^-sorted cells was in some cases sufficient for establishing a xenograft (Tables S2 and S3 in File S4), the overall rates of engraftment for CD133^+^-sorted cells were not significantly better than for bulk, unsorted cells (2/5 vs. 25/45, respectively). Given that both CD133^−^ and CD133^+^ cells from metastatic CRC tumors have the potential to establish xenografts [6], and, in order to capture the heterogeneity of the original human tumor as much as possible, the decision was made to use unsorted CRC tumor cells for xenotransplantation in all subsequent experiments. We also compared implantation of tumor cells under the kidney capsule with subcutaneous implantation, in view of published reports of high rates of engraftment of CRC cells after infra-renal capsule injection [1]. When simultaneous injections of equal numbers of CRC cells from the same tumor were made in the flank and under the kidney capsule of 3 different mice, the resulting subcutaneous tumors were far larger than those that developed in the kidney. Moreover, subcutaneous injection of cells in the flank was associated with a higher rate of xenograft establishment than subcapsular injection (3/14 vs. 26/45, *p* = 0.03 by two-tailed Fisher’s exact test) (Tables S2 and S3 in File S4), and was associated with less morbidity and early mortality, with early deaths at ≤1 week occurring in 14 of 30 mice that received subcapsular injections and 6 of 208 mice that received subcutaneous implants alone (*p* = 0.0001).

Based on these preliminary observations, subcutaneous injection of bulk, unsorted CRC cells into the flank was used in all subsequent experiments to establish CRC xenografts. The xenografts established in this fashion were retrospectively analyzed for characteristics that might be associated with preferred engraftment. There was no significant correlation between engraftment success and the clinical characteristics of the patients from whom tumors were obtained (Table S4 in File S4). Male patient gender was associated with a trend toward superior engraftment (*p* = 0.06). The mean number of cells injected in successful and failed engraftment attempts was significantly different –1.2×10^6^ (range: 2×10^5^–2×10^6^) cells in successful injections vs. 8.8×10^5^ (range: 1×10^4^–2×10^6^) in failed attempts (*p* = 0.04).

Serial xenografts were established from 18 of 26 first-generation, 11 out of 14 second-generation, and 8 out of 8 third-generation xenografts. Some xenografts were not passaged due to small size (≤1 mm in diameter), unexpected mouse death prior to tumor harvest, or the unavailability of recipient NSG mice. Of the 26 first-generation xenografts for which serial passaging was attempted, those that had demonstrated exponential growth showed a higher rate of engrafting in the second-generation than those that did not (16/20 vs. 2/6, respectively, *p* = 0.05), and none of the xenografts that failed to show exponential growth could be successfully passaged beyond the second generation. All of the xenograft lines that were passaged beyond the second generation yielded sizeable tumors >5 mm, and could grow to the maximum allowable tumor size of 2 cm. One primary colon tumor (D55949) and one metastatic tumor (P2726) were both serially transplanted through 10 generations. The amplification in human tumor cell number made possible by xenografting was in most cases at least 50-fold from the original cell dose for each generation, allowing for efficient and rapid expansion of CRC cells.

### Histology of CRC Tumors and their Derivative Xenografts

**Figure 1:**
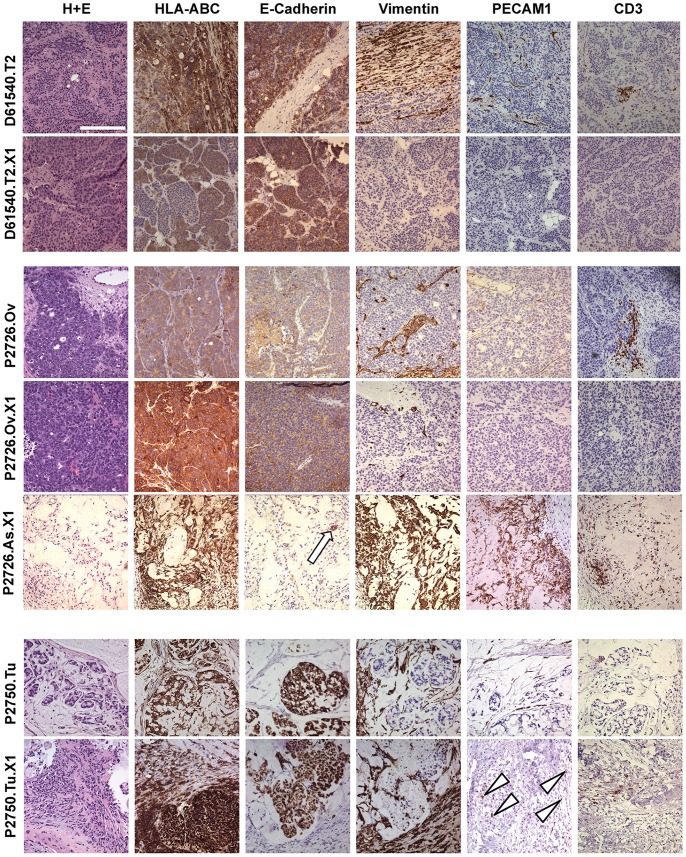
Histologic features of representative parental human CRC tumors and their derivative xenografts in NSG mice. Each row in (A)–(C) comprises micrographs of tissue sections from a single parental human tumor or murine xenograft. (A) Left column: tissue sections stained with hematoxylin and eosin (H+E). Subsequent columns, from left to right: tissue sections stained for human leukocyte antigen class I (HLA-ABC), epithelial marker E-cadherin, mesenchymal marker vimentin, endothelial marker PECAM1, and T-cell marker CD3. The rows, from top to bottom, show the histologic features of parental human tumors from D61540, P2726, and P2750, paired with their first-generation xenografts. The histology of the stroma-predominant xenograft developed from P2726 ascites fluid is also shown in the fifth row; the white arrow in the E-cadherin micrograph in this row indicates a small focus of tumor cells. Arrowheads in the PECAM1 micrograph for the P2750.Tu.X1 xenograft indicate areas with PECAM-1-immunoreactive cells. All images were obtained at 200x magnification. White scale bar in the upper left micrograph represents 200 µm.

Most first-generation xenografts demonstrated variably glandular morphology with well-defined epithelial and stromal components, resembling the parental human tumors (PHTs) from which they were derived (Figures 1 and Fig S1 Fig S2). Homogeneous expression of human class I major histocompatibility complex (MHC) molecules was observed in all CRC surgical specimens evaluated in this study, regardless of their derivation from primary tumors or from recurrent/metastatic lesions. Expression of human class I MHC in first-generation xenografts, however, showed two distinct patterns. All first-generation xenografts derived from primary colon tumors and all but three of those derived from recurrent/metastatic lesions showed homogeneous expression of human class I MHC in the epithelial components but absence of expression in the stromal components (Figures 1 and Fig S1 Fig S2), suggesting that the stroma in these xenografts was of murine origin, as has previously been reported [7], [9]. These xenografts will be referred to as carcinoma xenografts (CXs). In contrast, first-generation xenografts derived from three different patients (P2726, P2750, and P2825) were predominantly stromal in morphology and showed expression of human class I MHC in both their epithelial and stromal components, demonstrating that the stroma in these xenografts was at least partially comprised of human cells (Figures 1 and Fig S3). All three of these patients had peritoneal spread of their disease. These stromal xenografts (SXs) were derived from the peritoneal metastases of patients P2750 and P2825 and from the malignant ascites fluid of patient P2726, from whose ovarian metastasis multiple CX lineages with the typical human tumor/murine stroma composition were also generated. Immunohistochemical staining with antibodies specific for human vimentin, a stromal protein, confirmed the presence of abundant human vimentin in the three SXs, while staining with human E-cadherin-specific antibodies revealed that epithelial tumor cells comprised a small fraction of cells in these SXs (Figures 1 and Fig S3).

Immunohistochemistry (IHC) also demonstrated that the vasculatures of CXs and SXs, as with their stromal elements, were of divergent origin. A subset of parental human tumors paired with their first generation xenografts were examined for expression of human platelet endothelial cell adhesion molecule (PECAM-1; CD31), which is selectively expressed in human endothelial cells and subsets of hematopoietic cells. Immunoreactivity for human PECAM-1 was observed in all of the PHTs, as expected, but not in their derivative CXs. Both of the SXs, however, demonstrated expression of human PECAM-1, although the distribution of human PECAM-1-immunoreactive cells in the SXs was somewhat irregular and did not perfectly recapitulate the distribution of such cells that was typically observed in the PHTs (Figures 1 and Fig S3). The presence of human stroma in SXs but its absence from the CXs was mirrored by a similar pattern of human CD3 expression. IHC revealed that CXs did not, in general, contain any cells that stained with antibodies to human CD3 and CD8, consistent with the loss of human infiltrating CD3**^+^** and CD8**^+^** cells from their derivative xenografts. Somewhat surprisingly, both of the SXs, however, contained cells that expressed human CD3 and CD8 and were most likely residual human T cells that had been co-injected with the tumor cell inoculum, persisted in the SXs, and infiltrated the xenografts more extensively than TIL from the PHT (Figures 1 and Fig S3, and data not shown).

The histologic characteristics of secondary and subsequent-generation CXs were in general quite similar to those of the preceding generation CXs from which they were derived. The epithelial component of most CX lineages maintained robust expression of both EpCAM and CEA through serial transplantation, and the number and distribution of cells expressing Ki-67 likewise remained stable (Figure S2A). The stroma and vasculature of these CX lineages were consistently negative for expression of human class I MHC (Figure S2A), suggesting that they were stably supported by murine stroma and blood vessels.

### Global Transcriptional Analysis of Colorectal Tumors and Xenografts

**Figure 2:**
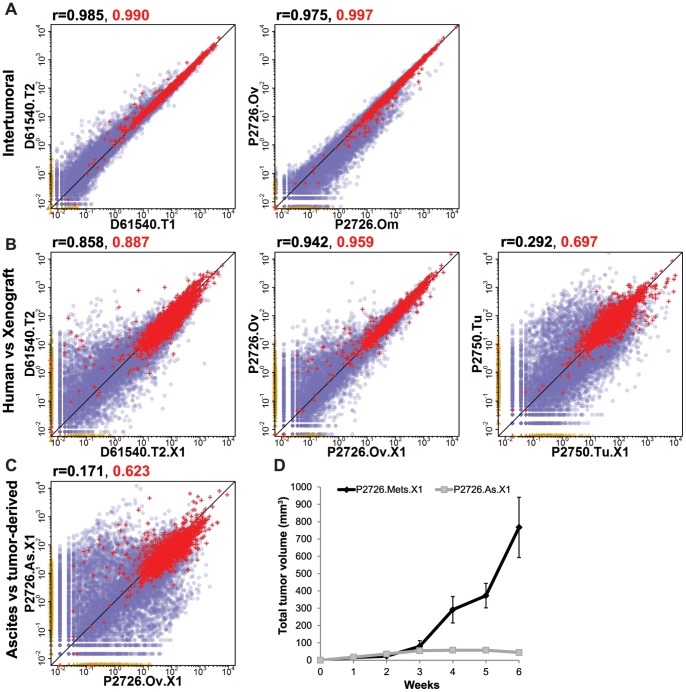
Pairwise comparison of expression of human genes in CRC tumors and derivative xenografts. Scatter plots display the normalized transcript counts (in counts per million) for individual human genes in the two samples that are indicated on the *x*- and *y*-axes. Red crosses indicate consensus housekeeping genes [50]. Non-housekeeping genes are indicated by violet circles or yellow triangles: violet circles represent genes expressed at some level in both samples, and yellow triangles along the axes represent genes expressed in one sample but not the other. The Pearson correlation coefficient across all genes for each pairwise comparison is indicated in black above the upper left corner of each plot; the correlation for the subset of housekeeping genes is indicated in red. The diagonal line is the locus of points for which x = y. (A) Comparisons of the expression level of human genes in the two halves of the bisected colorectal tumor from D61540 (left panel) and in the ovarian and omental metastases from P2726 (right panel). (B) Comparisons of the expression level of human genes in three parental human tumors (D61540.T2, P2726.Ov, and P2750.Tu, from left to right) and in their first-generation xenografts (D61540.T2.X1, P2726.Ov.X1, and P2750.Tu.X1). (C) Comparison of the human gene expression levels in the atypical stroma-predominant xenograft established from P2750 and in its first-generation xenograft. (D) Mean growth kinetics of CXs (n = 5) derived from the ovarian and omental metastases of P2726 (P2726.Mets.X1) are indicated by black diamonds connected by the bold line, compared with the growth kinetics of the SX derived from the ascites fluid of P2726 (P2726.As.X1) indicated by the gray squares connected by the gray line. All xenografts were established by injecting 2×10^6^ cells into the flank of a NSG mouse. Bars indicate standard error of the mean.

RNA-seq was used to define the human and, for xenografts, murine transcriptional profiles of 4 colon cancer samples –2 primary tumors and 2 metastatic tumors, 11 xenografts established from these tumor samples, and one sample of normal colon adjacent to one of the metastatic tumors (P2750) (Files S1 and S2). To assess the reproducibility of RNA-seq analysis of colon cancer samples and their derivative xenografts, we compared the transcriptional profiles of the two halves of a bisected primary colon tumor from patient D61540. This analysis revealed tight correlation (Pearson coefficient r = 0.985) between the expression levels of all human genes in the two halves of the sample (Figure 2A). A similarly tight correlation (r = 0.975) was observed between the transcriptional profiles of the synchronously resected but non-contiguous ovarian and omental metastases from patient P2726 (Figure 2A).

To define the global changes associated with the transition from human colon tumor to mouse xenograft, we compared the transcriptional profiles of three human colon tumors with those of their first-generation xenografts. Two of these were CXs (established from D61540 and P2726) and had the typical histologic pattern of human carcinoma supported by murine stroma, while the third (P2750) displayed the variant pattern of a SX. The transcriptomes of the first-generation xenografts established from D61540 and P2726 were reasonably similar to those of the parental tumors from which they were derived, with correlation coefficients of r = 0.858 and r = 0.942, respectively (Figure 2B). In contrast, a significantly higher degree of global similarity was observed between the transcriptomes of the first- and second-generation xenografts derived from the D61540 and P2726 PHTs, with r = 0.985 and r = 0.988, respectively (Figure S4A). Similar transcriptional stability, over seven generations, was seen in a xenograft lineage established by injection of CD133^+^ cells purified from the tumor of patient D55949 (Figure S4), which are enriched for tumor-initiating cells and depleted of stromal cells [1], [4].

In contrast to the xenografts established from D61540 and P2726, the transcriptional profile of the atypical (Figure 1) first-generation SX established from a peritoneal metastasis to the colon in P2750 was weakly correlated (r = 0.292) with the parental tumor from which it was derived (Figure 2B). Similarly, the transcriptional profile of the SX established from malignant ascites fluid of P2726 bore little resemblance to the human tumors or other xenografts derived from P2726 (Figure 2C). The SXs derived from P2726 ascites fluid and P2750 tumor could not be sustained as xenograft lines beyond the second generation (Table S2 in File S4), in contrast to the CXs derived from D61540 and the P2726 ovarian metastasis, both of which were serially transplanted through 10 generations. Moreover, the SXs did not increase significantly in size after the first week, in comparison to that of the CXs, which demonstrated progressive growth (Figure 2D, Table S2 in File S4).

The surface molecules CD133, CD166, CD44, and CD24, either alone or in combination, have been proposed as markers for CRC stem cells [1]–[3], [30]. We examined the expression of the corresponding genes – *PROM1*, *ALCAM*, *CD44*, and *CD24*, respectively – in PHTs and their derivative xenograft lineages to determine if serial xenografting was associated with changes in the expression of any of these genes (File S1). A trend toward higher levels of expression of *ALCAM*, *CD24*, and *CD44*, but not *PROM1*, was observed in CXs derived from D61540 when compared with their parental tumor. *ALCAM* and *CD44* were also expressed at higher levels in the two SXs from P2750 and P2726, when compared with the parental tumors from which the SXs were derived. No trend in the expression of these genes, however, was observed between the metastatic CRC sample from P2726 and the xenografts derived from it. Stably high levels of expression of all four genes, without a significant trend, were also observed in the xenograft lineage established from CD133-enriched cells from D55949 (File S1). Thus, RNA-seq analysis of the PHT/xenograft lineages in our series does not suggest that serial xenotransplantation is associated with consistent changes in the expression of *PROM1*, *ALCAM*, *CD44*, or *CD24*.

Comprehensive molecular profiling of colorectal cancer as part of The Cancer Genome Atlas initiative (19) has identified a set of genes that are recurrently mutated in colorectal tumors. We therefore specifically examined the sequence reads that mapped to a set of genes that are frequently mutated in colorectal cancer for evidence of genetic variants that have not previously been identified and classified as polymorphisms and might therefore represent tumor-specific mutations. A heterozygous C→T transition within APC not previously identified as a naturally occurring polymorphism or reported in the COSMIC mutation database (20) and predicted to create a premature termination codon in the APC coding sequence at position 861 was consistently observed in 62–82% of the reads from all samples in the D61540 tumor lineage, including the two halves of the parental tumor as well as its first- and second-generation xenografts (Table 2). Capillary sequencing of genomic DNA from the D61540 tumor, its xenografts, and from the normal adjacent colon tissue from this patient confirmed the heterozygous variant at position 861 in the parental tumor and xenografts, but revealed only the wild type APC sequence in the normal adjacent colon, suggesting that the variant seen in the tumor and xenografts was indeed a tumor-specific mutation. Likewise, a heterozygous G→T transition within TCF7L2 leading to a glutamine to lysine substitution at codon 51 was observed in both the parental tumor and the first- and second-generation xenografts with the mutant and wild type transcripts maintained at a consistent ratio (Table 2).

**Table 2:** Stability of mutated transcripts in xenografts.

Gene	Sample	Forward WT	Forward Mut	Reverse WT	Reverse Mut	% WT	% Mut
APC:Q861*	D61540.T1	0	3	1	1	20%	80%
APC:Q861*	D61540.T2	2	5	1	0	38%	62%
APC:Q861*	D61540.T2.X1	1	15	5	8	21%	79%
APC:Q861*	D61540.T2.X2	1	5	1	4	18%	82%
**TCF7L2:E51K**	P2726.Om	21	7	15	6	73%	27%
**TCF7L2:E51K**	P2726.Ov	13	13	15	18	47%	53%
**TCF7L2:E51K**	P2726.Ov.X1	6	7	6	8	44%	54%
**TCF7L2:E51K**	P2726.Ov.X2	1	5	6	4	44%	54%

The prevalence of a glutamine to stop codon mutation at the 861 amino acid position of *APC* (APC:Q861*) and a glutamate to lysine mutation at 51 amino acid position of *TCF7L2* were evaluated on the forward and reverse transcripts from the parental human tumors (gray rows) and first and second generation xenografts (white rows) of D61540 and P2726, respectively.

### Differential Expression Analysis

**Figure 3:**
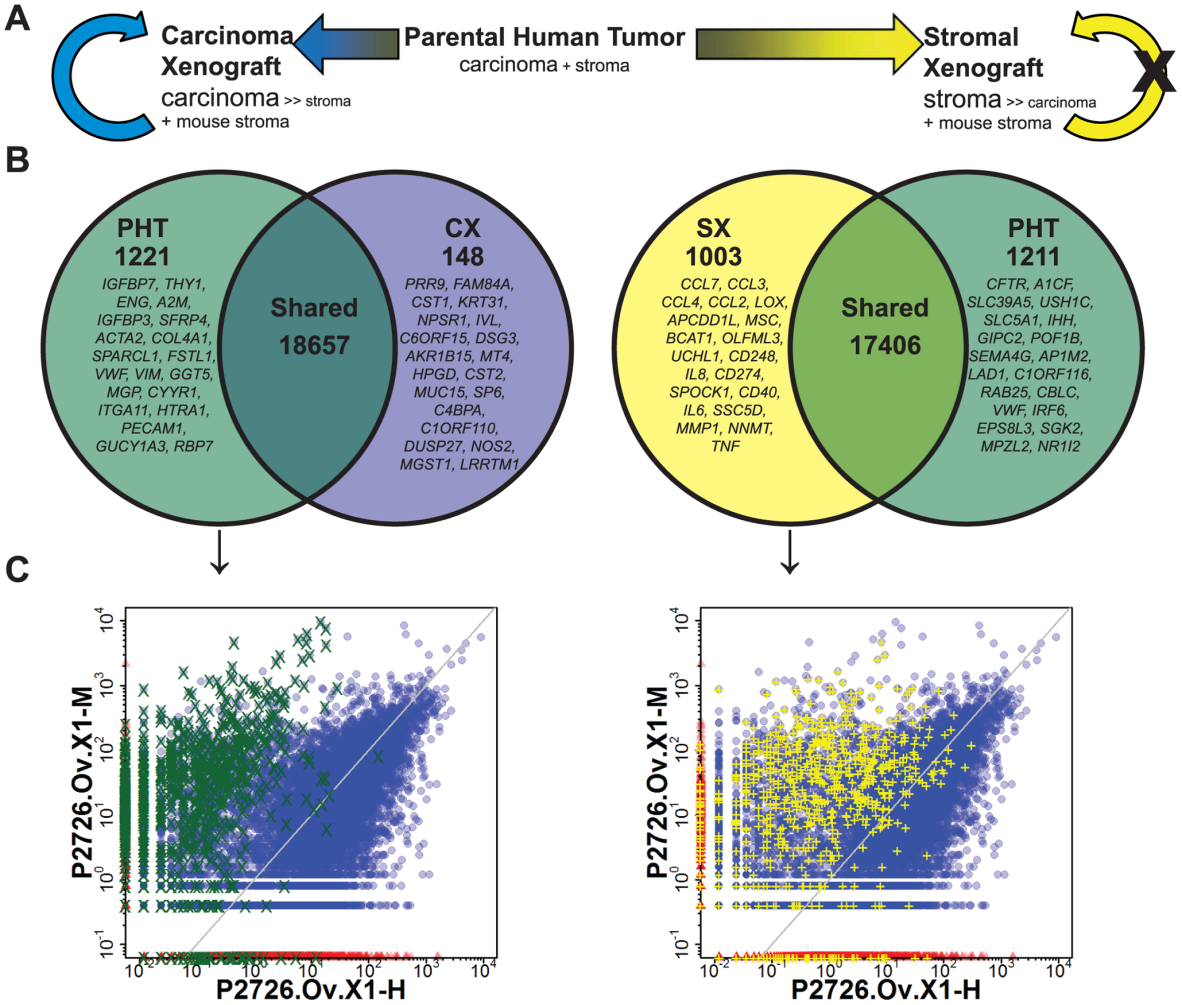
Differential expression analysis of human genes in parental tumors and derivative xenografts. (A) Conceptual model illustrating the bipartite classification of CRC xenografts as either Carcinoma Xenografts (CXs) or Stromal Xenografts (SXs). (B)Differential expression of all genes between the D61540.T1, D61540.T2, P2726.Om, and P2726.Ov parental human tumors (PHTs) and their derivative CXs D61540.T2.X1, D61540.T2.X2, P2726.Ov.X1, P2726.Ov.X2 (left Venn diagram), or between the P2726.Om, P2726.Ov, and P2750 PHTs and their derivative SXs P2726.As.X1 and P2750.Tu.X1 (right Venn diagram). The number following the “X” in each xenograft name indicates its passage generation. The number of genes with a Benjamini-Hochberg false discovery rate (FDR) of 0.05 or higher, which were interpreted as being “shared,” is indicated in the area of intersection in each Venn diagram. The number of genes with FDR <0.05, which are candidates for differentially expressed genes, are indicated under the labels PHT, CX, or SX. Listed in each Venn diagram are the 20 human genes with the lowest FDR. (C) Relative expression of orthologous human/murine genes in a representative xenograft. The symbols indicate the normalized human (*x* axis) and murine (*y* axis) transcript counts for genes with human and murine orthologues in the first generation xenograft from the P2726 ovarian CRC metastasis (P2726.Ov.X1). In the left plot, green ‘X’s indicates the read counts for genes that were preferentially expressed in SXs over PHTs (SX >PHT), and in the right plot yellow ‘+’s indicate the read counts for genes that were preferentially expressed in PHTs over CXs (PHT>CX). The read counts for all other orthologous human/murine gene pairs are indicated by blue circles for genes expressed in both and red triangles for genes expressed in one but not the other. The gray diagonal line represents 1∶1 correlation.

Given the observation that all but two of the first-generation xenografts in our series displayed the pattern of human tumor cells supported by murine stroma and vasculature, we wanted to determine the total number and identity of human genes whose expression was significantly down-regulated in the transition from parental tumor to xenograft. We also wanted to determine whether the reliance on a murine rather than human stroma would be associated with prominent changes in the expression of human genes in the tumor cells in xenografts. Differential expression analysis was performed to determine the number and identity of genes whose expression levels changed significantly during the transition from PHT to CX or SX (Figure 3). A total of 1369 genes were differentially expressed in the D61540 and P2726 PHT/CX pairs, with 1221 expressed at a lower level in the xenografts and 148 at a higher level (Figure 3B). As expected, the transcriptomes of these 2 xenografts were distinguishable from their parental tumors by the near- or complete absence of expression of human stromal genes such as fibronectin (*FN1*), vimentin (*VIM*), endosialin (*CD248*), and several stroma-specific collagen genes and matrix metallopeptidases (*COL1A2, COL6A2*, *COL6A3*, *MMP2*, and *MMP9*). The first-generation xenografts from D61540 and P2726 also showed no significant expression of human *PECAM1* (*CD31*) or genes that encode components of the CD3 complex (*CD3D*, *CD3E*, and *CD3G*), confirming the histological observation that these xenografts contained solely murine endothelium and lacked human T cells.

A total of 2214 genes were differentially expressed between the P2726.Ov and P2750 PHTs and their derivative SXs, with 1003 expressed at a higher level in the SXs and 1211 at a lower level (Figure 3B). Unlike the typical CXs established from D61540 and P2726.Ov PHT, the SXs from P2726 and P2750 expressed high levels of human stromal genes such as *FN1*, *VIM*, *CD248*, *MMP2*, and *MMP9*; stromal collagen genes such as *COL1A2, COL6A2*, *COL6A3*; and a high level of human *PECAM1*, consistent with the abundant human stroma and frequent human CD31^+^ endothelial cells noted in these tumors on histology (Figure 1). Expression of CD3 genes – *CD3D*, *CD3E*, and *CD3G*, as well as the CD8 α chain – *CD8A* – was clearly present in the SXs, as would be expected from the histologic identification of human CD3**^+^** and CD8*^+^* cells in the SXs (Figures 1). Differential expression analysis also revealed increased expression levels of several inflammatory and immunomodulatory cytokines and signaling molecules (File S3).

Gene set enrichment analysis (GSEA) of the genes that showed significant differential expression between PHTs and SXs or CXs using the KEGG, Reactome, and Biocarta gene categories within MSigDB revealed that pathways for cell adhesion, hematopoietic and immune signaling, and G-protein coupled receptor signaling were amongst the most significantly over-represented (File S3). The majority of the genes in these sets were preferentially expressed in SXs over PHTs and in PHTs over CXs, reflecting the relative abundance of stromal, epithelial, and hematopoietic cells in each sample type.

Alignment of the RNA-seq data obtained from CXs and SXs to the human and murine genomes (File S1) confirmed the anticipated differences in gene expression between the human and murine compartments of the two types of xenografts. The murine orthologues of many of the differentially expressed human stromal genes were expressed at high levels in the CXs, consistent with the conclusion drawn from histological analysis that the stroma in these xenografts was of murine origin. Indeed, there is extensive and consistent overlap between the genes that were preferentially expressed in SXs over PHTs and in PHTs over CXs, with the genes that were preferentially expressed in the murine compartments of CXs (Figure 3C). This phenomenon is also evident in the transcriptomes of the second-, third-, fourth-, and seventh-generation xenografts derived from patient D55949 (Table S5 in File S4). Thus, the murine compartment of CXs largely recapitulates the transcriptomic signature of the original human tumor stroma.

**Figure 4:**
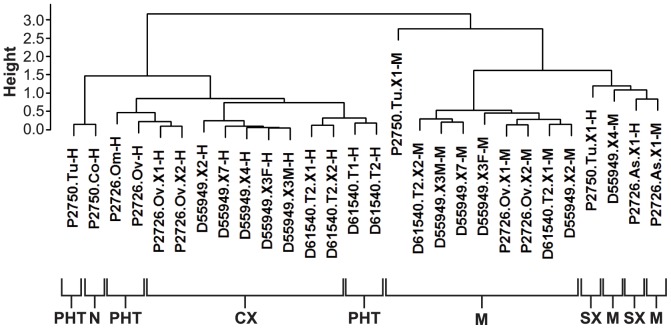
Cluster analysis of mouse and human orthologues in sequenced samples. Pearson correlation was performed between all samples, and Euclidean distances were calculated. Unsupervised hierarchical cluster analysis of the human and murine transcriptomes, defined across 17,179 orthologous human-mouse gene pairs, in all of the parental tumors and derivative xenografts in which RNA-seq analysis was then performed. The height of the vertical dendrogram arms correlates with the global difference in the transcriptomes between any two samples. The height of the vertical dendrogram arms is proportional to the global differences in gene expression between samples in a cluster group. Along the bottom of the panel, samples are coded as follows: N = normal colon, PHT = parental human tumor, SX = stromal xenograft, CX = carcinoma xenograft, M = mouse stroma. Human and murine transcriptomes are indicated by ‘-H’ and ‘-M’ suffixes, respectively.

Unsupervised hierarchical cluster analysis of the orthologous mouse and human gene transcriptomes in all of the PHTs and xenografts was performed to evaluate the extent to which global similarity between specific parental tumor-xenograft lineages could be identified. This analysis revealed, as expected, that the human transcriptomes in xenografts clustered by lineage, and that all CX lineages clustered in turn with the parental human tumors from which they were derived (Figure 4). The human transcriptomes of the atypical SXs derived from P2750 and the ascites fluid of P2726, however, did not cluster closely with the transcriptomes of their parental tumors, nor did they cluster closely with those from any of the other xenograft lineages (Figure 4). The human transcriptomes of the 2 SXs clustered more closely with the murine transcriptomes of the xenografts. Moreover, the murine transcriptomes in the xenografts did not cluster according to their respective human lineages (Figure 4), suggesting that the expression profile of the murine component of each xenograft is not strongly influenced by the transcriptome of the human tumor cells in that xenograft.

### Xenografts Maintain Expression of Immunotherapy Target Genes

**Figure 5:**
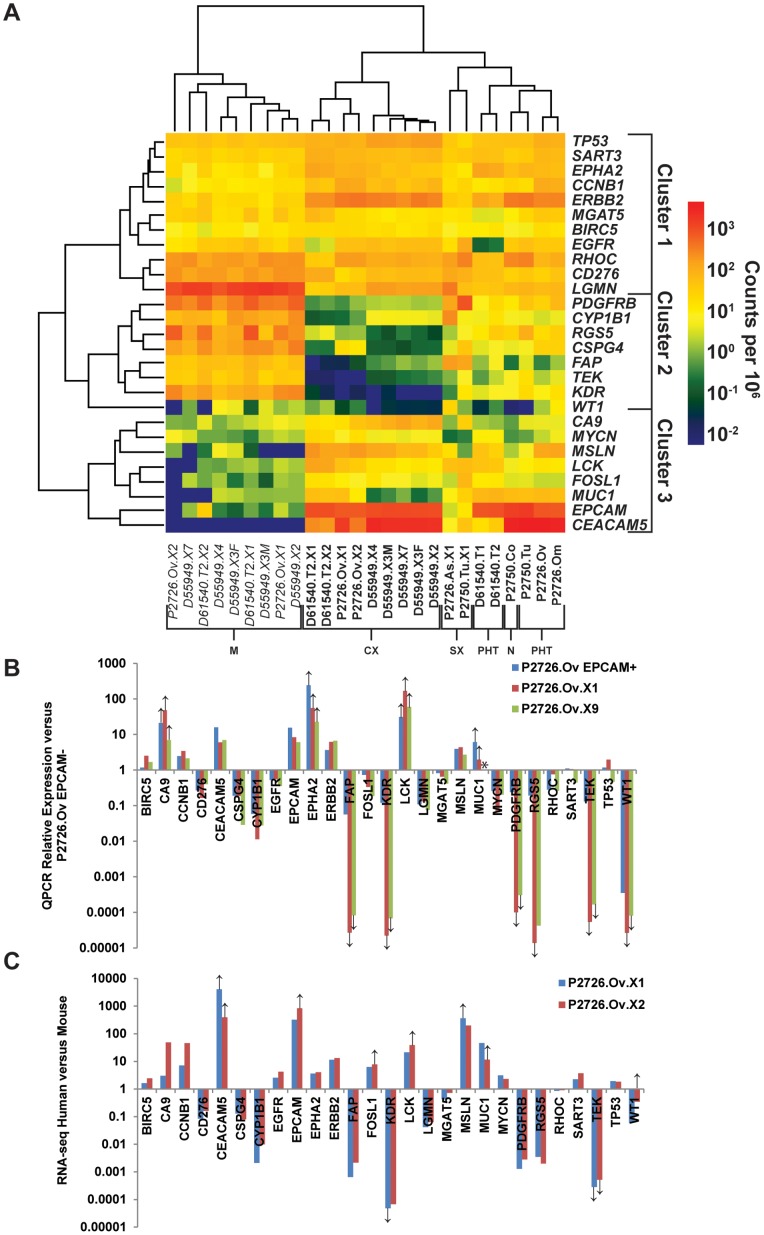
Expression of genes encoding high-priority cancer immunotherapy targets in parental tumors and xenografts. (A) Expression of genes encoding high priority immunotherapy targets in parental tumors or xenografts (indicated by bold text along bottom), or of their murine orthologues in xenografts (indicated by italic text along bottom), is shown in a log-transformed heatmap, with unsupervised hierarchical cluster analysis performed on all samples (*x*-axis) and genes (*y*-axis). The total height of the vertical dendrogram arms separating any two samples is proportional to the differences in gene expression across all listed genes between those samples. The scale bar correlates normalized read counts to color. Along the bottom of the panel, samples are coded as follows: N = normal colon, PHT = parental human tumor, SX = stromal xenograft, CX = carcinoma xenograft, M = mouse stroma. (B) Ratio of expression of immunotherapy target genes in EPCAM^+^ cells from the P2726.Ov PHT and its derivative first- and ninth-generation xenografts to their expression in EPCAM^−^ cells from the P2726.Ov PHT. Expression was determined by real-time PCR and LinRegPCR, and normalized to housekeeping gene *GAPDH*. Down or up arrows indicate genes for which the minimal expression values have been substituted for undetectable transcript in the EPCAM^+^ or EPCAM^−^ fractions, respectively. The asterisk indicates a comparison for which both samples did not have detectable transcript. (C) Ratio of expression of human immunotherapy target genes and their murine orthologues, as inferred from RNA-seq data, in the first- and second-generation xenografts from the P2726.Ov. Down or up arrows indicate genes for which the minimum human or murine read counts, respectively, in each sample were substituted for read counts of 0.

Stability of gene expression is requisite for the efficacy of targeted treatments such as immunotherapy. We thus examined the expression in PHTs and xenografts of a subset of the 75 human genes previously prioritized by the National Cancer Institute for cancer vaccine development [31]. Unsupervised cluster analysis revealed 3 patterns of expression: (1) genes ubiquitously expressed amongst PHTs, CXs, SXs, and mouse stroma (Figure 5A, cluster 1); (2) a subset of genes, typified by *FAP, PDGFRB*, *CYP1B1*, and *RGS5,* that were expressed in the PHTs and SXs but not in the CXs, and whose murine orthologues were expressed at high levels in CXs, suggesting that they were primarily expressed in the stroma (Figure 5A, cluster 2); and (3) genes such as human *EPCAM* and *CEACAM5* that were expressed at high levels in the PHTs and all of the CXs, but at lower levels in the SXs and in the murine transcriptome of CXs, suggesting that they were selectively expressed in carcinoma cells (Figure 5A, cluster 3).

The expression of the selected subset of immunotherapy target genes was further evaluated by real-time PCR in sorted stromal (EPCAM^−^) and carcinoma (EPCAM^+^) cell populations from the P2726.Ov PHT and in EPCAM**^+^** human cells from the first and ninth generation CXs of this tumor (Figure 5B). For the majority of genes in clusters 1 and 3, the level of expression measured in the EPCAM**^+^** cells in the PHT was comparable to that observed in the EPCAM**^+^** (human) compartment of the first and the ninth generation CXs. In contrast, the predominantly stromal genes in cluster 2 were expressed at high levels in EPCAM^−^ cells of the PHT but at much lower levels in the first and ninth generation CXs. The differences in the expression of these genes between the EPCAM**^+^** and EPCAM^−^ components of the PHT (Figure 5B) were qualitatively consistent with the differences in gene expression demonstrated by RNA-seq between the human carcinoma and murine stromal components of P2726-derived xenografts (Figure 5C). These real-time PCR data are consistent with those of the RNA-seq analysis, and suggest that the human compartment of CXs only expresses the subset of immunotherapy target genes that are expressed in the EPCAM**^+^** but not EPCAM^−^ cells of the parental tumor.

### Aberrant Expression of Hematopoietic Genes in CRC Cells

**Figure 6:**
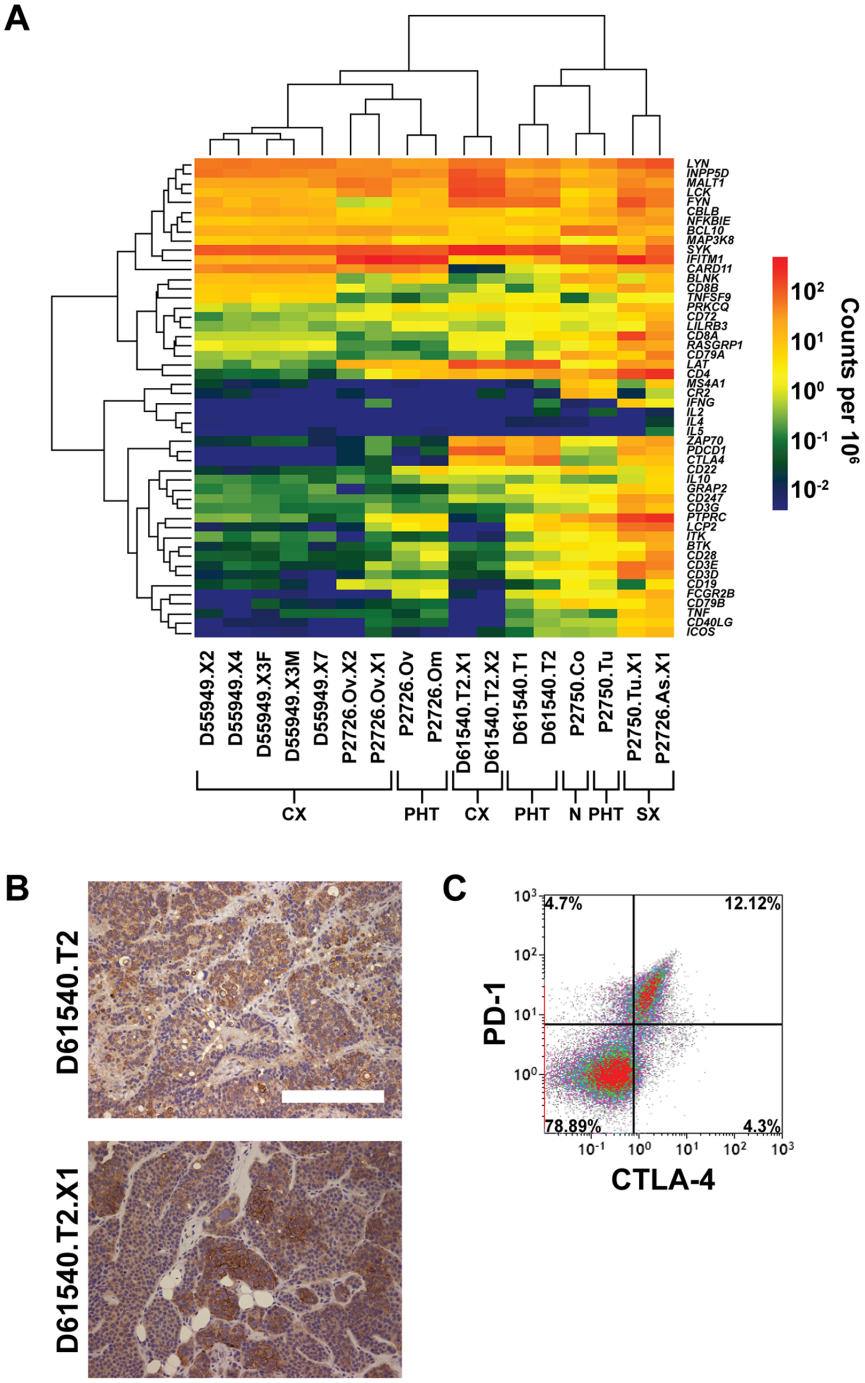
Expression of human B and T cell receptor signaling-associated genes in tumors and xenografts. (A) Expression of genes associated with B or T cell receptor signaling are shown in the log-transformed heatmap with unsupervised hierarchical cluster analysis performed on all samples (*x*-axis) and genes (*y*-axis). The total height of the vertical dendrogram arms separating any two samples is proportional to the differences in gene expression across all listed genes between those samples. The scale bar correlates normalized read counts to color. Along the bottom of the panel, samples are coded as follows: N = normal colon, PHT = parental human tumor, SX = stromal xenograft, CX = carcinoma xenograft. (B) Immunohistochemical staining for PDCD1 in the D61540 parental tumor (top) and its first-generation xenograft (bottom). White scale bar in top micrograph represents 200 µm. (C) Flow cytometric analysis of human PDCD1 (PD-1) and CTLA-4 expression by cells isolated from the D61540 first-generation xenograft.

The presence of human CD3**^+^** and CD8**^+^** T cells in SXs but their absence from CXs (Figure 1) led to the expectation that human genes that are normally expressed only in T lymphocytes and other hematopoietic cells would be detectable in the two SXs in our series, but negligible in the balance of CXs. RNA-seq analysis of the two SXs confirmed expression of a broad array of human lymphocyte and hematopoietic genes (data not shown). Differential expression analysis (File S2) was then used to determine which, if any, of these genes were specifically down regulated in CXs. *CD45* (*PTPRC*), expressed by all hematopoietic cells except erythrocytes and plasma cells, *CD20* (*MS4A1*), a marker of B cells, and many B and T cell receptor-associated signaling molecules such as *BLNK*, *BTK*, *FCGR2B*, *CD79B*, *CD28*, and *LCP2*, were indeed expressed at significantly lower levels in the CXs when compared with their PHTs (Figure 6A). Surprisingly, however, despite the histologic evidence of the absence of human B and T cells within the CXs, the expression of many genes in B- and T-cell signaling pathways was not diminished in these xenografts (Figure 6A). The expression of several lymphocyte signaling molecules, including *CTLA4*, *LAT*, *PDCD1*, and *ZAP70*, was maintained in the transition from human tumor to CX, and was particularly prominent in the CXs derived from D61540 (Figure 6). IHC confirmed that PD-1 protein (PDCD-1) was heterogeneously expressed on tumor cells in both the D65140 PHT and its first-generation CX (Figure 6B). Moreover, flow cytometry revealed that PD-1 and CTLA-4 were mostly co-expressed in tumor cells harvested from the D61540 CX (Figure 6C), suggesting that expression of these two T cell-associated inhibitory signaling molecules in CRC cells may be coordinately regulated. Expression of *PD-L1* and *PD-L2*, both of which encode ligands for PD-1 and are frequently expressed on the surface of tumor cells, was low in both the PHT and CX from D61540 (File S1).

## Discussion

Xenotransplantation of human CRC in immune-deficient mice is being used with increasing frequency both to dissect the biology of this common cancer and to evaluate therapeutic approaches. Patient-derived CRC xenografts have been established by injecting purified populations of cells in which tumor-initiating capacity is thought to reside [1]–[4], [6], or even isolated single tumor stem cells [8], as well as by injecting unsorted cell suspensions prepared from tumor samples [9], as we have. Regardless of method, injection of human CRC cells into immune-deficient mice can reproducibly establish xenografts that faithfully recapitulate the morphologic heterogeneity and molecular diversity of the original human tumor, and such xenografts represent a promising model system for the study of targeted therapeutics against CRC. Studies with patient-derived CRC xenografts have reproduced and confirmed the clinical resistance or sensitivity to cetuximab therapy according to *KRAS* mutation status that was observed in clinical trials [9], and selection of chemotherapy-resistant clones has also been observed in CRC xenografts [10]. These studies have suggested that patient-derived CRC xenografts will have utility for answering mechanistic questions and for guiding efficient development of clinical therapeutics [32].

In agreement with the results of other reported series of patient-derived CRC xenografts [1]–[17], we found that xenografts were readily established from the majority of, but not all, CRC surgical specimens, and that samples of primary CRC could be established as readily as those from metastatic CRC tumors. We observed during the course of this study that our success rate for xenograft establishment steadily improved, suggesting that, in experienced hands, the true proportion of CRC samples that can be successfully xenografted is likely to be significantly higher than the overall success rate reported for this study (27 of 50; 54%). Nonetheless, there remains a subset of CRC samples that cannot readily be xenografted. Determining the reasons for failure in these cases – which could potentially be linked to clinical correlates – and revising experimental procedures to overcome these limitations, represent important research priorities.

Most of the CRC xenografts established in our series comprised human tumor cells supported by murine stroma. The mixed human/murine composition of these CXs was established by histology and extensively confirmed by deep transcriptional analysis with RNA-seq, which allowed us to determine unambiguously the human or murine origin of >97% of sequence reads with a high degree of confidence similar to what has been reported with another bi-species alignment algorithm for RNA-seq data from xenografts [33]. In our series, the replacement of almost all human stroma and vasculature with murine analogues was clearly observed in first generation CXs in contrast to studies from other investigators reporting that human stroma and vasculature could be retained in early generations [15], [34] but replaced over time by murine components. The reason for the immediate loss of human stroma in our xenografts may be due to the fact that our tumors were mostly dissociated to single cell suspension prior to implantation in the mouse, rather than implanted as a single piece of tissue, which may diminish the capacity of the human stroma to persist.

Nonetheless, despite the single cell suspension technique, we also generated from 3 patients with peritoneal metastases histologically atypical “stromal” xenografts that contained abundant human stroma and blood vessels; the human origin of the stroma in these xenografts was documented both histologically and by transcriptional analysis with RNA-seq. The transcriptomes of 2 SXs did not closely resemble those of the parental tumors from which they were derived. The unique features of the SXs cannot solely be attributed to their origin from peritoneal metastases, as tumor cells from the ovarian and omental metastases in P2726 were able to generate CXs as well as SXs. Moreover, in contrast to the majority of CXs in our series that were readily propagated via serial transplantation – in 2 cases out to 10 generations – we were unable to propagate SXs beyond 2 generations. Another notable distinction between the CXs and the 3 SXs, which was again strongly supported by transcriptional analysis of the two types of xenografts, was the unequivocal presence of human CD3**^+^** and CD8**^+^** T cells in the latter but their absence from the former. These observations suggest that CD3**^+^**CD8**^+^** tumor infiltrating lymphocytes (TIL), which were present in the cell suspensions from which our xenografts were established (J. Chou and E.H. Warren, *manuscript in preparation*), can persist in CRC xenografts that contain human stromal elements. It is possible that these TIL interfere with or inhibit continued propagation of SXs, or alternatively that other types of cells derived the stroma in the parental tumor interfere with their propagation.

Transcriptional analysis of PHTs and their derivative xenograft lineages revealed that the PHT to 1° xenograft transition is characterized by significant changes in gene expression, most of which reflect and are, in fact, due to the systematic replacement of human stroma and vasculature in most xenografts by murine analogues. Once established as a xenograft, however, patient-derived CRC cells remain transcriptionally stable for at least seven generations, and likely longer. They continue to express class I MHC, components of the class I and class II MHC antigen processing and presentation pathways, and potential tumor-specific genes for immunotherapy at levels comparable to those observed in the PHTs. Recently published studies [9], [15] similarly concluded that the transcriptomes of patient-derived CRC cells remained transcriptionally stable with serial xenografting, but this conclusion was based on results obtained with microarrays designed specifically for analysis of human but not murine transcriptomes. Unsupervised cluster analysis of the transcriptional profiles defined with human microarrays in that study demonstrated, as might be anticipated, that the transcriptomes of all the primary human tumors clustered most closely with each other, and that all the transcriptomes of the xenografts likewise clustered most closely with each other [9]. In contrast, our transcriptional analysis using RNA-seq found that the transcriptomes of parental tumors and xenografts clustered by lineage, with individual xenografts clustering most closely with the parental human tumors from which they were derived. We believe that our RNA-seq-based analysis constitutes a far more stringent evaluation of transcriptional fidelity in patient-derived CRC xenografts than analysis with microarrays designed specifically for the human transcriptome.

The absence of human stromal cells from patient-derived CRC xenografts is quite crucial given the increasingly important role that tumor stroma is felt to play in the biology and therapeutic response of human CRC. It is unclear whether the absence of human stroma from the vast majority of CRC xenografts is attributable to properties that are intrinsic to the CRC tumor cells, to properties intrinsic to the human stromal cells in the parental tumor, or both. Mesenchymal stem cells (MSCs), the progenitors of stromal cells, have been found to have both anti-tumor and tumor-promoting effects in gastrointestinal cancers [35], and the development of therapy targeting specific components of CRC stroma, such as fibroblast activation protein produced by tumor-associated fibroblasts [36], [37], is an active area of research. The tumor stroma is an important target during T cell-mediated tumor rejection in murine models [38], [39], and ongoing studies in our lab suggest that some CRC TIL are specifically reactive with the stromal elements in autologous tumors (J. Chou and E.H. Warren, *manuscript in preparation*). Thus, the value of patient-derived xenografts for therapeutic studies of CRC may be profoundly enhanced if methods can be developed for establishing xenografts that contain human stroma but also faithfully retain the transcriptional profile of their parental tumors. Conversely, experimental therapies targeting the analagous mouse stroma in CRC xenografts may also be investigated given its similarity to the human stroma. In contrast to patient-derived CRC xenografts, xenografts of many human glioblastomas contain human endothelial cells that are derived from progenitor cells in the malignant population [34], [40], and comparative analysis of human glioblastoma and CRC may provide insight into the characteristics that are responsible for this difference.

The expression of select T- and B-cell associated signaling genes has previously been described in human CRC. Over-expression of *LCK* and cytotoxic T cells specific for LCK-encoded peptides have been described in poor prognosis colon cancers [41], [42]. *SYK* over-expression has been described in other solid tumors, such as breast and gastric cancer, and seems to correlate with improved prognosis and reduced invasion [43]. Expression of *NKG2D*, which is primarily expressed on natural killer and CD8**^+^** T cells, has also been observed in CRC cells, but was not observed in any of the tumors in our series [44]. Expression of the inhibitory signaling molecule PD-1 is usually limited to T cells, while its ligands PD-L1 and PD-L2 are found on a subset of tumors, including CRC [45], [46]. Inhibition of PD-1-mediated signaling with monoclonal antibodies has been associated with regression of several different types of solid tumors, but clinical trials of antibodies that interrupt signaling through the PD-1/PD-L1 axis in patients with CRC have to date shown limited benefit [46], [47]. We observed expression of *PDCD1* (which encodes PD-1) and another T cell inhibitory receptor, *CTLA4*, in a subset of tumor cells in the PHT and CXs from D61540, and confirmed co-expression of their protein products on the surface of tumor cells by flow cytometry. While it is possible that CRC cells may utilize certain T- or B-cell signaling molecules to promote their own proliferation, it is also unclear what survival advantage T cell-inhibitory receptors would offer CRC cells, and whether they would respond negatively or positively to antibody-mediated interference in these inhibitory pathways. A possible mechanism by which CRC cells may acquire expression of lymphocyte-associated genes is through fusion with lymphocytes, which has long been hypothesized as a mechanism for metastasis [48] and has been observed between intestinal tumors and macrophages *in vivo* [49].

In conclusion, our results suggest that a majority of primary and metastatic CRC samples can be propagated serially as xenografts NSG mice. These xenografts closely resemble their parental human tumors both transcriptionally and phenotypically, with the important exceptions that the human stroma and vasculature in the PHTs are systematically replaced by murine analogues in their derivative xenografts, and most or all of the infiltrating lymphocytes from the parental tumor are also lost. Xenografts that retain human stroma, vasculature, and lymphocytes are maintained can be established from a small minority of CRC samples, but serial transplantation of such xenografts has not been possible to date. Elucidation of the factors that permit initial establishment of stromal xenografts, and those that inhibit their serial propagation, are the focus of ongoing studies. Serially transplantable CRC xenograft lines offer a reliable system for expanding tumor cells from patients for experimental study of novel targeted therapies such as immunotherapy.

## Supporting Information

Figure S1 **Histologic characteristics of representative CRC colon tumors and their first-generation xenografts.** Tissue sections from the D55959 and D61211 parental colon tumors and their first-generation xenografts. were stained with H+E or with human-specific antibodies to HLA-ABC, EpCAM, E-Cadherin, Vimentin, or PECAM1. White scale bar in the upper left micrograph represents 200 µm.
(TIF)

Figure S2 **Histologic characteristics of representative CRC liver metastases and their first-generation xenografts.** Tissue sections from P2762 and P2792 parental liver metastases and their first-generation xenografts were stained with H+E or with human-specific antibodies to HLA-ABC, EpCAM, E-Cadherin, Vimentin, or PECAM1. White scale bar in the upper left micrograph represents 200 µm.
(TIF)

Figure S3 **Histologic characteristics of a CRC peritoneal metastasis and its first-generation xenograft.** Tissue sections from the P2825 omental metastasis and its first generation xenograft were stained with H+E or with human-specific antibodies to HLA-ABC, EpCAM, E-Cadherin, Vimentin, PECAM1, or CD3. White scale bar in the upper left micrograph represents 200 µm.
(TIF)

Figure S4 **Histologic and transcriptional stability of human CRC xenografts through serial transplantation.** (A) Columns, from left to right: micrographs of tissue sections of the D55949 parental tumor (top row), 1° xenograft (middle row), and 7° xenograft (third row) stained with hematoxylin and eosin (H+E) or with antibodies specific for HLA-ABC, EpCAM, carcinoembryonic antigen (CEA), or Ki-67. All images were obtained at 200x magnification. White scale bar in the upper left micrograph represents 200 µm. (B) Comparison of the expression of human genes in the 1°, 2°, 3° (male and female), and 7° xenografts in the D55949 lineage and the 1° and 2° xenografts in the P2726 lineage. Each symbol indicates the normalized transcript counts for an individual human gene in the two samples indicated on the *x*- and *y*-axes. The Pearson correlation coefficient for each pairwise comparison is indicated in the upper left corner of each plot. The symbol indicating *HBB,* the only differentially expressed gene identified in any of the comparisons, is indicated by the text label in the middle panel of the bottom row.
(TIF)

File S1 **RNA-seq human transcripts (counts per million).**
(XLS)

File S2 **Counts of sequence reads aligned to Mm9, with annotation of differentially expressed genes.**
(XLS)

File S3 **Gene set enrichment analysis using MSigDB.**
(XLS)

File S4 **Tables S1–S5. Table S1. Primer pairs for real-time PCR analysis of expression of immunotherapy target genes. File S1.** Counts of sequence reads aligned to Hg19, with annotation of differentially expressed genes. **Table S2. Characteristics of patients from whom CRC xenografts were established.** The last successful serial passage of a xenograft line is indicated for each parental human tumor. **Bold** = injected subcutaneously; Underline = injected under kidney capsule; *Italics* = CD133 sorted; VU = CHTN - Vanderbilt University; UW = University of Washington; M = male; F = female; Mod. = moderate; MSS = microsatellite stable; MSI = microsatellite instability; MMRD/P = mismatch repair deficient/proficient; WT = wild type; mut = mutant; † = determined from RNA-seq data; LVI = lymphovascular invasion; Ind. = indeterminate; Tx = received chemotherapy prior to resection; # cells implanted = number of cells implanted from parental human tumor; Non exp. = R^2^<0.5 when tumor growth data fitted to an exponential growth curve; + = successfully xenografted without failure; * = did not attempt passage; ⊗ = could not continue serial xenografts. **Table S3. Characteristics of patients from whom CRC xenografts could not be established.** **Bold** = injected subcutaneously; Underline = injected under kidney capsule; *Italics* = CD133 sorted; VU = CHTN - Vanderbilt University; UW = University of Washington; M = male; F = female; Mod. = moderate; MSS = microsatellite stable; MSI = microsatellite instability; MMRD/I = mismatch repair deficient/intact; WT = wild type; mut = mutant; LVI = lymphovascular invasion; Tx = received chemotherapy prior to resection; Alive >1 wk = mice injected with CRC survived longer than 1 week. **Table S4. Association of tumor characteristics with engraftment.** Various characteristics of CRC tumors from which bulk tumor cells were subcutaneously implanted into NSG mice were evaluated to determine whether they associated with successful engraftment or not. P-values were determined by two-tailed Fisher’s exact test. UW = University of Washington; VU = CHTN, Vanderbilt University; * = primary versus metastatic site of tumor, which includes synchronously resected primary and metastatic tumors from 3 patients; LVI = lymphovascular invasion. **Table S5. Distribution of differentially expressed gene sets between mouse and human transcriptomes.** Genes preferentially expressed in parental human tumors over carcinoma xenografts (PHT>CX) or in stromal xenografts over parental human tumors (SX >PHT), indicated in Figure 3C, were evaluated for whether they were expressed predominantly as mouse or human orthologues in each xenograft. Only genes with mouse and human orthologues were evaluated.
(DOCX)
