# Supplementary figures and images for: Phenotypic and Transcriptional Fidelity of Patient-Derived Colon Cancer Xenografts in Immune-Deficient Mice
Source: PLoS One. 2013 Nov 20;8(11):e79874. doi: 10.1371/journal.pone.0079874 (PMC3835935; doi:10.1371/journal.pone.0079874)

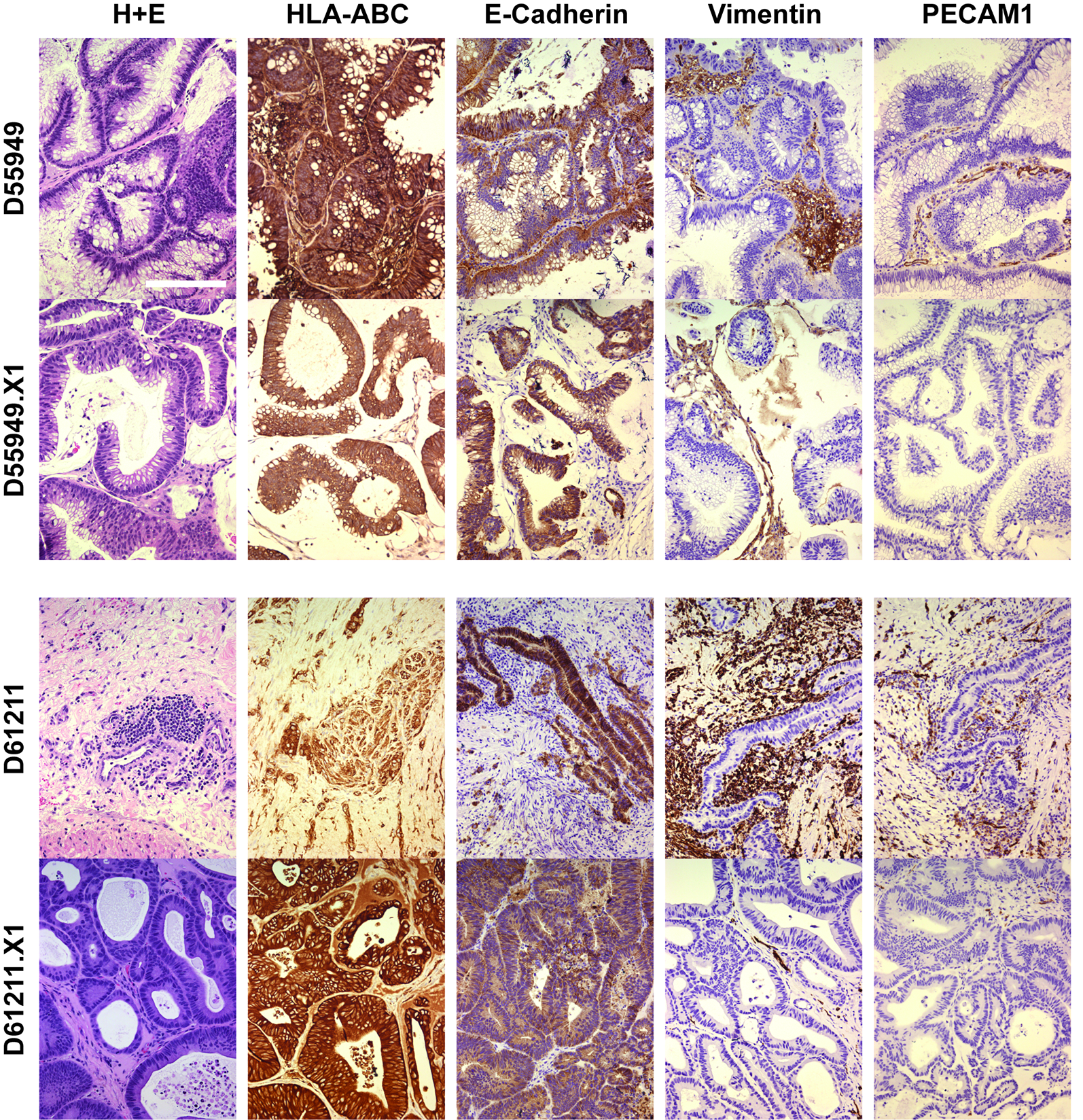

Supplement: Figure S1 — Histologic characteristics of representative CRC colon tumors and their first-generation xenografts. Tissue sections from the D55959 and D61211 parental colon tumors and their first-generation xenografts. were stained with H+E or with human-specific antibodies to HLA-ABC, EpCAM, E-Cadherin, Vimentin, or PECAM1. White scale bar in the upper left micrograph represents 200 µm. (TIF) [file pone.0079874.s001.tif]

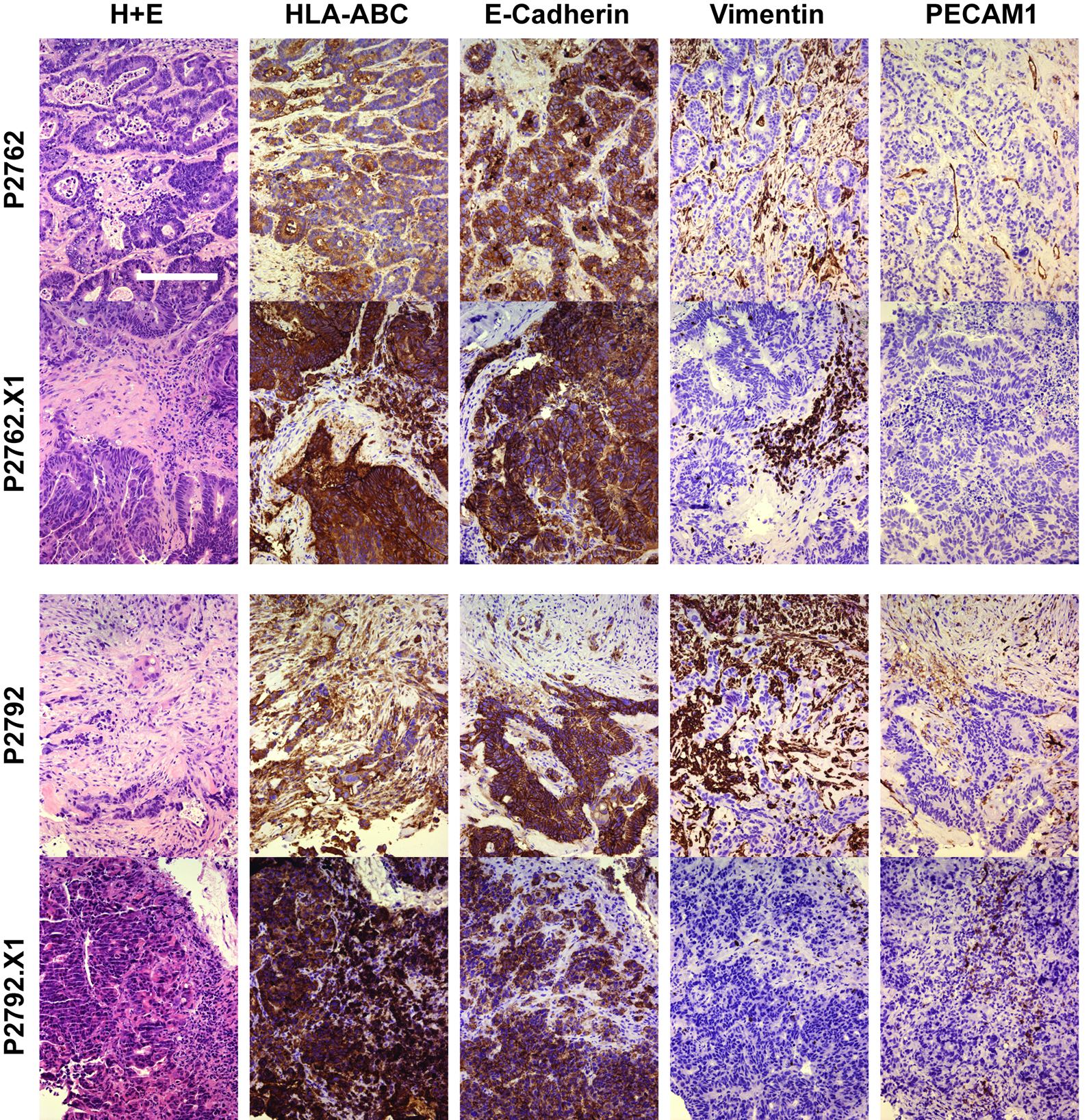

Supplement: Figure S2 — Histologic characteristics of representative CRC liver metastases and their first-generation xenografts. Tissue sections from P2762 and P2792 parental liver metastases and their first-generation xenografts were stained with H+E or with human-specific antibodies to HLA-ABC, EpCAM, E-Cadherin, Vimentin, or PECAM1. White scale bar in the upper left micrograph represents 200 µm. (TIF) [file pone.0079874.s002.tif]

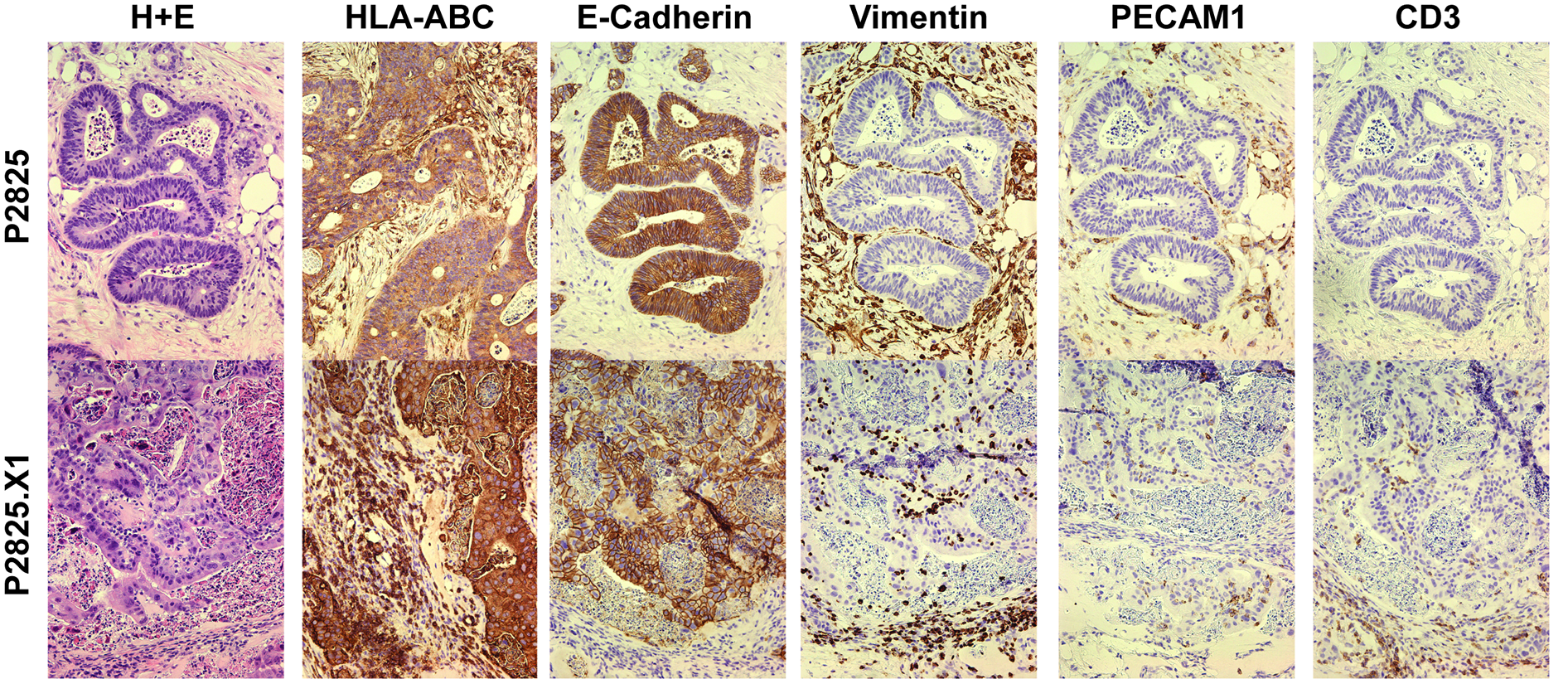

Supplement: Figure S3 — Histologic characteristics of a CRC peritoneal metastasis and its first-generation xenograft. Tissue sections from the P2825 omental metastasis and its first generation xenograft were stained with H+E or with human-specific antibodies to HLA-ABC, EpCAM, E-Cadherin, Vimentin, PECAM1, or CD3. White scale bar in the upper left micrograph represents 200 µm. (TIF) [file pone.0079874.s003.tif]

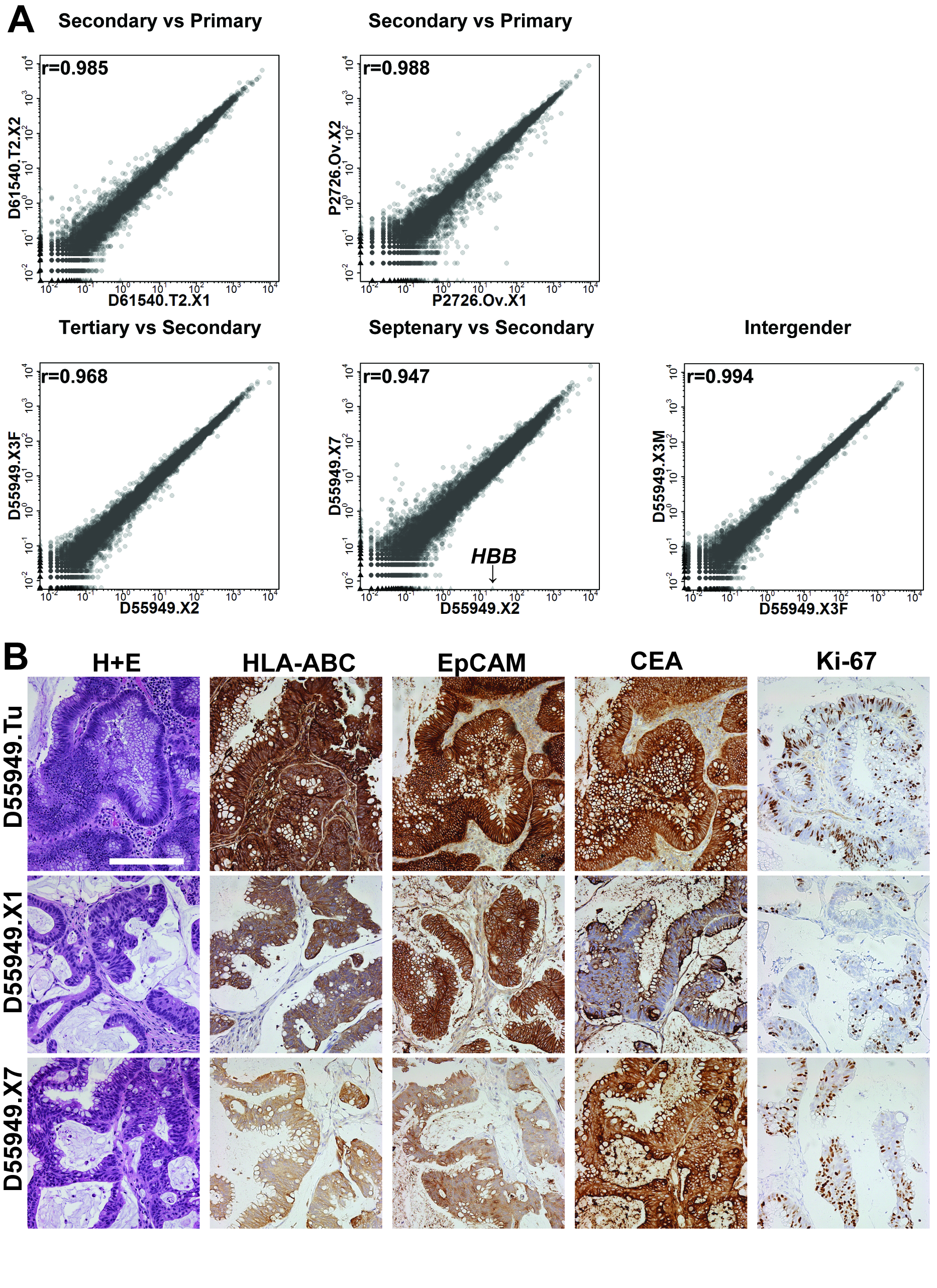

Supplement: Figure S4 — Histologic and transcriptional stability of human CRC xenografts through serial transplantation. (A) Columns, from left to right: micrographs of tissue sections of the D55949 parental tumor (top row), 1° xenograft (middle row), and 7° xenograft (third row) stained with hematoxylin and eosin (H+E) or with antibodies specific for HLA-ABC, EpCAM, carcinoembryonic antigen (CEA), or Ki-67. All images were obtained at 200x magnification. White scale bar in the upper left micrograph represents 200 µm. (B) Comparison of the expression of human genes in the 1°, 2°, 3° (male and female), and 7° xenografts in the D55949 lineage and the 1° and 2° xenografts in the P2726 lineage. Each symbol indicates the normalized transcript counts for an individual human gene in the two samples indicated on the x- and y-axes. The Pearson correlation coefficient for each pairwise comparison is indicated in the upper left corner of each plot. The symbol indicating HBB, the only differentially expressed gene identified in any of the comparisons, is indicated by the text label in the middle panel of the bottom row. (TIF) [file pone.0079874.s004.tif]
