# Supplementary material for: Phenotypic and Transcriptional Fidelity of Patient-Derived Colon Cancer Xenografts in Immune-Deficient Mice
Source: PLoS One. 2013 Nov 20;8(11):e79874. doi: 10.1371/journal.pone.0079874 (PMC3835935; doi:10.1371/journal.pone.0079874)
Supplement: File S4 — Tables S1–S5. Table S1. Primer pairs for real-time PCR analysis of expression of immunotherapy target genes. File S1. Counts of sequence reads aligned to Hg19, with annotation of differentially expressed genes. Table S2. Characteristics of patients from whom CRC xenografts were established. The last successful serial passage of a xenograft line is indicated for each parental human tumor. Bold = injected subcutaneously; Underline = injected under kidney capsule; Italics = CD133 sorted; VU = CHTN - Vanderbilt University; UW = University of Washington; M = male; F = female; Mod. = moderate; MSS = microsatellite stable; MSI = microsatellite instability; MMRD/P = mismatch repair deficient/proficient; WT = wild type; mut = mutant; † = determined from RNA-seq data; LVI = lymphovascular invasion; Ind. = indeterminate; Tx = received chemotherapy prior to resection; # cells implanted = number of cells implanted from parental human tumor; Non exp. = R2<0.5 when tumor growth data fitted to an exponential growth curve; + = successfully xenografted without failure; * = did not attempt passage; ⊗ = could not continue serial xenografts. Table S3. Characteristics of patients from whom CRC xenografts could not be established. Bold = injected subcutaneously; Underline = injected under kidney capsule; Italics = CD133 sorted; VU = CHTN - Vanderbilt University; UW = University of Washington; M = male; F = female; Mod. = moderate; MSS = microsatellite stable; MSI = microsatellite instability; MMRD/I = mismatch repair deficient/intact; WT = wild type; mut = mutant; LVI = lymphovascular invasion; Tx = received chemotherapy prior to resection; Alive >1 wk = mice injected with CRC survived longer than 1 week. Table S4. Association of tumor characteristics with engraftment. Various characteristics of CRC tumors from which bulk tumor cells were subcutaneously implanted into NSG mice were evaluated to determine whether they associated with successful engraftment or not. P-values were determined [file pone.0079874.s008.docx]

**Table S1. Primer pairs for real-time PCR analysis of expression of immunotherapy target genes**

| Gene | 5’ primer | 3’ primer |
| --- | --- | --- |
| *BIRC5* | GTTGCGCTTTCCTTTCTGTC | TCCGCAGTTTCCTCAAATTC |
| *CA9* | CCTTTGCCAGAGTTGACGA | CTCAGCGATTTCTTCCAAGC |
| *CCNB1* | TACCTATGCTGGTGCCAGTG | AGATGTTTCCATTGGGCTTG |
| *CD276* | GGAGAATGCAGGAGCTGAGG | AGCTGTAGGGAGGGGTAGC |
| *CEACAM5* | GCCAAAATCACGCCAAA | CCAGCTGAGAGACCAGGAGA |
| *CSPG4* | TTGGCTTTGACCCTGACTATG | CTGCAGGTCTATGTCGGTCA |
| *CYP1B1* | AACGTACCGGCCACTATCAC | CCACGACCTGATCCAATTCT |
| *EGFR* | AAACAACACCCTGGTCTGGAAG | TCTTAGGCCCATTCGTTGGA |
| *EPCAM* | TGGAATTGTTGTGCTGGTTATTTC | GTGTCCATTTGCTATTTCCCTTC |
| *EPHA2* | GTGCAGTGGATGGCGAGT | GGCTCTCAGATGCCTCAAAC |
| *ERBB2* | GCCTGCCACCCCTGTT | CACAGCCACCGGCACA |
| *FAP* | GCTGTGCTTGCCTTATTGGT | TCAGTGTGAGTGCTCTCATTGTAT |
| *FOSL1* | GGGCATGTTCCGAGACTTC | TGACTGCCACTCATGGTGTT |
| *KDR* | CTCCCGAGTTCTGGGCATT | AGCCTGGGCAGATCAAGAGA |
| *LCK* | GATCTCACAATCTCAGGGACCAT | GAGCCATTTCGGATGAGCA |
| *LGMN* | GAAGATTCGGACGTGGAAGA | GTTTCCATACTGCATGACGTG |
| *MGAT5* | ACCGGAACAAACTCAACCAA | AGTGAGGGTAGCCGTCCATA |
| *MSLN* | ATTTGAAGGCGCTCAGTCAG | CCAGAAGTTTCTGCACCTCAG |
| *MUC1* | GGTTCTGGTCATGCAAGCTCTAC | GAGACCCCAGTAGACAAAGCA |
| *MYCN* | CAGTCGGCGGGAGTGTT | TCCGCCCCGTTCGTT |
| *PDGFRB* | TGTCCAGAGCCTGGAACTGT | GCCCTGAGAGATCTGTGGTT |
| *RGS5* | GTGCAAAGGACTTGCAGCTT | GGTCTTGGCTGGTTTCTCTG |
| *RHOC* | CGGAAGCCTTGACTTCATCT | CACCAGCTTCTTTCGGATTG |
| *SART3* | CGAGTGGGAATATGACGAAGA | CATCCTCACCTTGGTAAGCTC |
| *TEK* | CTTGGCAACATATTCAAGTGACAAA | AAGCGTCTCACAGGTCCA |
| *TP53* | GCTTTCCACGACGGTGAC | GCTCGACGCTAGGATCTGAC |
| *WT1* | CGCTATTCGCAATCAGGGTTA | CCTCATGCTTGAATGAGTGGTT |
| *GAPDH* | CTCTGCTCCTCCTGTTCGAC | TTAAAAGCAGCCCTGGTGAC |

**Table S2. Characteristics of patients from whom CRC xenografts were established**

| Subject | Site | Age | Sex | Pathology | Grade | Genetics | Location | Stage | LVI | Tx | # cells implanted | Doubling time (days) | Xenograft generations |
| --- | --- | --- | --- | --- | --- | --- | --- | --- | --- | --- | --- | --- | --- |
| *D49708* | VU | 63 | F | Adenocarcinoma | Poor | - | R colon | T3N2b | Yes | No | 1x10^4^-1x10^6^ | - | 1* |
| D52955 | VU | 72 | M | Adenocarcinoma | Mod. | - | R colon | T3N0 | No | No | 1x10^6^ | 35.9 | 1* |
| *D55949* | VU | 49 | M | Adenocarcinoma with mucinous features | Mod. | K-ras mut†, BRAF WT† | R Colon | T3N2b | No | No | 1x10^4^-1x10^5^ | 7.3 | 10+ |
| D51638 | VU | 69 | F | Adenocarcinoma with mucinous features | Poor | - | R colon | T3N0 | No | No | 1x10^6^ | 10.1 | 1⊗ |
| D52751 | VU | 47 | M | Adenocarcinoma with mucinous features | Mod. | - | R colon | T3N1bM1a | Yes | No | 1x10^6^ | 16.2 | 1⊗ |
| D60739 | VU | 46 | F | Adenocarcinoma | Mod. | - | Sigmoid | T3N1bM1a | Yes | No | 1x10^6^-2x10^6^ | 7.0 | 3+ |
| P9625 | UW | 36 | F | Adenocarcinoma | Mod. | MSS | R colon | T3N1b | Yes | No | 1x10^6^ | Non-exp. | 1* |
| D61211 | VU | 80 | M | Adenocarcinoma | Mod. | - | Sigmoid | T3N0 | No | No | 6x10^5^ | 5.3 | 3+ |
| D61540 | VU | 83 | M | Adenocarcinoma with minor neuroendocrine component | Poor | K-ras mut†, BRAF WT† | R Colon | T3N1b | Yes | No | 1x10^6^ | 12.1 | 7+ |
| P2726 | UW | 41 | F | Metastatic adenocarcinoma | - | MSS, K-ras WT, BRAF WT† | Ovary,  Omentum,  Ascites | M1b | - | Yes | 1x10^6-^2x10^6^ | 5.7,  5.9,  Non-exp. | Ovary-10+, Omentum-1+, Ascites-1⊗ |
| P2750 | UW | 26 | M | Mucinous adenocarcinoma | - | MMRP, K-ras WT, BRAF WT† | R colon-peritoneal | M1b | - | Yes | 1x10^6^ | Non-exp. | 2⊗ |
| P2762 | UW | 55 | M | Metastatic adenocarcinoma | - | - | Liver | M1a | - | Yes | 1x10^6^ | 9.4 | 4+ |
| P2792 | UW | 49 | F | Metastatic adenocarcinoma | - | MMRP, K-ras mut | Liver | T4aN2aM1a | - | Yes | 1x10^6^ | 7.8 | 6+ |
| P2773 | UW | 51 | F | Metastatic adenocarcinoma | - | K-ras mut | Retro-peritoneal | M1b | - | Yes | 2x10^5^ | 8.6 | 5+ |
| WD2112 | VU | 50 | F | Adenocarcinoma | Mod. | MSS, K-ras WT | L colon | T3N2a | No | No | 1x10^6^-2x10^6^ | 11.3 | 2+ |
| WD2713 | VU | 78 | F | Adenocarcinoma | Poor | MSI | R colon | T3N0 | No | No | 1x10^6^-2x10^6^ | Non.exp. | 2⊗ |
| P2797 | UW | 66 | F | Adenocarcinoma | Well | MSS | Recto-sigmoid | T3N0 | No | No | 1x10^6^ | Non-exp. | 1⊗ |
| P2796 | UW | 45 | M | Neuroendocrine | Well | - | Rectum | T3N1bM1b | Ind. | No | 6x10^5^-1x10^6^ | Non-exp. | 1⊗ |
| P2807 | UW | 63 | M | Metastatic adenocarcinoma | - | K-ras WT, BRAF WT | Liver | M1a | - | Yes | 3x10^5^ | Non-exp. | 1* |
| P2808 | UW | 66 | F | Metastatic adenocarcinoma | - | K-ras mut, BRAF WT | Lung | M1a | - | Yes | 1x10^6^ | 9.4 | 5+ |
| P2816 | UW | 52 | M | Metastatic adenocarcinoma | - | K-ras WT, BRAF WT | Liver | M1a | - | No | 1x10^6^ | 9.4 | 4+ |
| P2818 | UW | 61 | M | Metastatic adenocarcinoma | - | K-ras mut | Liver | M1a | - | Yes | 1x10^6^ | 20.6 | 3+ |
| P2822 | UW | 64 | M | Adenocarcinoma | Mod. | - | Sigmoid | T3N2a | Yes | No | 2x10^6^ | 34.0 | 2+ |
| P2824 | UW | 66 | M | Adenocarcinoma | Mod. | - | Sigmoid,  Liver | T3N0M1a | Ind. | Yes | 1x10^6^ | 47.2,  138.6 | Sigmoid-1⊗, Liver-2⊗ |
| P2825 | UW | 64 | M | Metastatic adenocarcinoma | - | K-ras mut, BRAF WT | Omentum, Abdominal wall | M1b | - | Yes | 5x10^5^-2x10^6^ | 24.7,  Non-exp. | Omentum-1⊗, Abd. wall-1⊗ |
| P2827 | UW | 78 | M | Adenocarcinoma with mucinous features | Well | - | R colon,  Liver | T3N2aM1a | Ind. | Yes | 1x10^6^-2x10^6^ | 9.3,  19.1 | Colon-2+, Liver-2+ |
| P2842 | UW | 54 | F | Adenocarcinoma | Well | MMRP, K-ras mut | Rectum,  Liver | T1N0M1a | No | Yes | 2x10^6^ | 7.3,  8.9 | Rectum-1+, Liver-1+ |

The last successful serial passage of a xenograft line is indicated for each parental human tumor. **Bold** = injected subcutaneously; Underline = injected under kidney capsule; *Italics* = CD133 sorted; VU = CHTN - Vanderbilt University; UW = University of Washington; M = male; F = female; Mod. = moderate; MSS = microsatellite stable; MSI = microsatellite instability; MMRD/P = mismatch repair deficient/proficient; WT = wild type; mut = mutant; † = determined from RNA-seq data; LVI = lymphovascular invasion; Ind. = indeterminate; Tx = received chemotherapy prior to resection; # cells implanted = number of cells implanted from parental human tumor; Non exp. = R^2^ < 0.5 when tumor growth data fitted to an exponential growth curve; + = successfully xenografted without failure; * = did not attempt passage; ⊗ = could not continue serial xenografts

**Table S3. Characteristics of patients from whom CRC xenografts could not be established.**

| Subject | Site | Age | Sex | Pathology | Grade | Genetics | Location | Stage | LVI | Tx | # cells implanted | Alive > 1 wk |
| --- | --- | --- | --- | --- | --- | --- | --- | --- | --- | --- | --- | --- |
| P3802 | UW | 55 | M | Metastatic adenocarcinoma | - | - | Liver | M1a | - | Yes | 1x10^5^ | Yes |
| P4816 | UW | 66 | F | Metastatic adenocarcinoma | - | ^-^ | Liver | M1a | - | Yes | 1x10^4^-2x10^6^ | No |
| P8172 | UW | 56 | F | Metastatic adenocarcinoma | - | K-ras mut | Liver | M1a | - | Yes | 5x10^5^ | Yes |
| D56241 | VU | 75 | M | Adenocarcinoma | Mod. | - | Cecum | T3N0 | No | No | 2x10^5^ | Yes |
| *D46208* | VU | 50 | M | Adenocarcinoma | Mod. | - | L colon | T3N1 | No | No | < 10^4^ | Yes |
| *D56242* | VU | 67 | M | Adenocarcinoma | Mod. | - | Rectum | T3N0 | No | No | 1x10^5^-5x10^5^ | Yes |
| *D49849* | VU | 73 | F | Adenocarcinoma with focal mucin production | Mod. | - | R colon | T3N0 | No | No | 1x10^4^ | Yes |
| D50147 | VU | 47 | M | Medullary carcinoma | Poor | ^-^ | Cecum | T3N1 | Yes | No | 1x10^6^ | No |
| D50473 | VU | 89 | F | Adenocarcinoma | Mod | ^-^ | R colon | T4bN0 | No | No | 1x10^6^ | Yes |
| D51646 | VU | 79 | F | Mucinous adenocarcinoma | Mod. | ^-^ | Sigmoid | T4bN0 | No | No | 5x10^5^ | Yes |
| P1892 | UW | 49 | F | Adenocarcinoma | Well | MMRD | Transverse colon | T3N0 | No | No | 5x10^5^ | No |
| D57405 | VU | 62 | F | Adenocarcinoma | Mod. | - | Recto-sigmoid, liver | T3N2aM1a | Yes | No | 3mm piece | No |
| P8948 | UW | 49 | F | Metastatic adenocarcinoma | - | MSS | Liver | M1a | - | Yes | 1x10^6^ | No |
| P7654 | UW | 37 | M | Metastatic adenocarcinoma | - | MSS, K-ras WT | Liver | M1a | - | Yes | 1x10^4^ | Yes |
| P7028 | UW | 68 | F | Metastatic adenocarcinoma with mucinous features | - | K-ras mut | Omentum | M1b | - | Yes | 3x10^4^ | Yes |
| P1854 | UW | 70 | F | Adenocarcinoma | Well | - | Rectum | T3N1 | No | Yes | 3x10^5^ | Yes |
| D60877 | VU | 77 | M | Adenocarcinoma | Mod. | - | L colon | T3N2a | No | No | 1x10^6^ | No |
| D61121 | VU | 52 | F | Adenocarcinoma | Mod. | MSS | L colon | T4aN2b | Yes | No | 1x10^6^ | Yes |
| D61187 | VU | 84 | F | Adenocarcinoma | Mod. | MSS | Rectum | T3N2b | No | No | 1x10^6^ | Yes |
| P1442 | UW | 59 | M | Adenocarcinoma | Well | K-ras WT | Sigmoid | T3N2bM1a | No | Yes | 3x10^5^ | Yes |
| D61462 | VU | 64 | F | Adenocarcinoma | Mod. | - | Sigmoid | T3N1a | No | No | 1x10^6^ | Yes |
| WD349 | VU | 70 | F | Adenocarcinoma with medullary features | Poor | MSI | R colon | T2N1c | No | No | 1x10^6^ | Yes |
| P2820 | UW | 65 | F | Metastatic mucinous adenocarcinoma | - | - | Liver | M1a | - | Yes | 1x10^6^ | Yes |

**Bold** = injected subcutaneously; Underline = injected under kidney capsule; *Italics* = CD133 sorted; VU = CHTN - Vanderbilt University; UW = University of Washington; M = male; F = female; Mod. = moderate; MSS = microsatellite stable; MSI = microsatellite instability; MMRD/I = mismatch repair deficient/intact; WT = wild type; mut = mutant; LVI = lymphovascular invasion; Tx = received chemotherapy prior to resection; Alive > 1 wk = mice injected with CRC survived longer than 1 week.

**Table S4. Association of tumor characteristics with engraftment**

| Characteristic | Condition | Success | Fail | Percentage | P-value |
| --- | --- | --- | --- | --- | --- |
| Site | UW | 17 | 7 | 71% | 0.19 |
|  | VU | 8 | 9 | 47% |  |
| Age | >60 | 13 | 10 | 57% | 0.54 |
|  | <60 | 12 | 6 | 67% |  |
| Sex | M | 14 | 4 | 78% | 0.06 |
|  | F | 11 | 12 | 48% |  |
| Mucinous | Yes | 3 | 3 | 50% | 0.66 |
|  | No | 22 | 13 | 63% |  |
| Primary vs metastatic^*^ | Primary | 15 | 12 | 56% | 0.35 |
|  | Metastasis | 13 | 5 | 72% |  |
| LVI (primary only) | Yes | 5 | 3 | 63% | 0.67 |
|  | No | 7 | 9 | 44% |  |
| Location (primary only) | Colon | 9 | 9 | 50% | 1.00 |
|  | Rectum | 3 | 3 | 50% |  |
| Received chemotherapy | Yes | 12 | 6 | 67% | 0.54 |
|  | No | 13 | 10 | 57% |  |

The characteristics of human CRC tumors from which bulk tumor cells were subcutaneously implanted into NSG mice were evaluated to determine whether they were associated with successful engraftment or not. P-values were determined by two-tailed Fisher’s exact test. UW = University of Washington; VU = CHTN, Vanderbilt University; * = primary versus metastatic site of tumor, which includes synchronously resected primary and metastatic tumors from 3 patients; LVI = lymphovascular invasion.

**Table S5. Distribution of differentially expressed gene sets between mouse and human transcriptomes.**

| Xenograft | Mouse only | Mouse > Human | Human only | Human > Mouse | Neither |
| --- | --- | --- | --- | --- | --- |
| PHT > CX | | | | | |
| D61540.T2.X1 | 243 (22%) | 665 (61%) | 85 (8%) | 12 (1%) | 81 (7%) |
| D61540.T2.X2 | 292 (27%) | 615 (56%) | 74 (7%) | 8 (1%) | 99 (9%) |
| P2726.Ov.X1 | 206 (19%) | 618 (57%) | 109 (10%) | 7 (1%) | 146 (13%) |
| P2726.Ov.X2 | 248 (23%) | 486 (45%) | 140 (13%) | 6 (1%) | 206 (19%) |
| D55949.X2 | 238 (22%) | 606 (56%) | 95 (9%) | 38 (3%) | 109 (10%) |
| D55949.X3F | 284 (26%) | 624 (57%) | 64 (6%) | 42 (4%) | 72 (7%) |
| D55949.X3M | 253 (23%) | 587 (54%) | 94 (9%) | 40 (4%) | 112 (10%) |
| D55949.X4 | 360 (33%) | 536 (49%) | 54 (5%) | 34 (3%) | 102 (9%) |
| D55949.X7 | 227 (21%) | 571 (53%) | 112 (10%) | 35 (3%) | 141 (13%) |
| SX > PHT | | | | | |
| D61540.T2.X1 | 87 (10%) | 581 (66%) | 78 (9%) | 87 (10%) | 41 (5%) |
| D61540.T2.X2 | 96 (11%) | 583 (67%) | 77 (9%) | 76 (9%) | 42 (5%) |
| P2726.Ov.X1 | 50 (6%) | 607 (69%) | 125 (14%) | 45 (5%) | 47 (5%) |
| P2726.Ov.X2 | 75 (9%) | 525 (60%) | 134 (15%) | 41 (5%) | 99 (11%) |
| D55949.X2 | 97 (11%) | 590 (68%) | 65 (7%) | 53 (6%) | 69 (8%) |
| D55949.X3F | 128 (15%) | 610 (70%) | 45 (5%) | 49 (6%) | 42 (5%) |
| D55949.X3M | 95 (11%) | 594 (68%) | 78 (9%) | 41 (5%) | 66 (8%) |
| D55949.X4 | 159 (18%) | 567 (65%) | 52 (6%) | 49 (6%) | 47 (5%) |
| D55949.X7 | 73 (8%) | 594 (68%) | 85 (10%) | 38 (4%) | 84 (10%) |

Genes preferentially expressed in parental human tumors over carcinoma xenografts (PHT > CX) or in stromal xenografts over parental human tumors (SX > PHT), indicated in Figure 3C, were evaluated for whether they were expressed predominantly as mouse or human orthologues in each xenograft. Only genes with mouse and human orthologues were evaluated.
